# Resveratrol ameliorates iron overload induced liver fibrosis in mice by regulating iron homeostasis

**DOI:** 10.7717/peerj.13592

**Published:** 2022-06-08

**Authors:** Hua Wang, Chuan Jiang, Yakun Yang, Jinghan Li, Yihan Wang, Chaonan Wang, Yonggang Gao

**Affiliations:** 1 Hebei University of Chinese Medicine, Department of Preventive Medicine, Shijiazhuang, Hebei, China; 2 Hebei University of Chinese Medicine, School of Pharmacy, Shijiazhuang, Hebei, China; 3 Hebei University of Chinese Medicine, Collge of Basic Medicine, Shijiazhuang, Hebei, China; 4 Hebei Key laboratory of Chinese Medicine Research on Cardio-Cerebrovascular Disease, Shijiazhuang, Hebei, China

**Keywords:** Resveratrol, Iron overload, Liver fibrosis, Antioxidant, Iron homeostasis

## Abstract

This study is intended to explore the protective effects of resveratrol (RES) on iron overload-induced liver fibrosis and its mechanism. Iron dextran (50 mg/kg) was injected intraperitoneally in all groups except the control group. Mice in the L-RES, M-RES and H-RES groups were gavaged with RES solution at 25, 50 mg/kg and 100 mg/kg, respectively, 4 h before injection of iron dextran every day; mice in the deferoxamine (DFO) group were injected with DFO intraperitoneally (100 mg/kg); mice in the control group received isovolumetric saline. After seven weeks of RES administration, serum alanine aminotransferase (ALT), aspartate aminotransferase (AST) activities and liver hydroxyproline (Hyp) levels were reduced; the malondialdehyde (MDA) activities decreased and the levels of superoxide dismutase (SOD) and glutathione (GSH) were raised. Hematoxylin and eosin (H&E), Prussian, and Masson staining indicated that RES treatment could improve cell damage and reduce hepatic iron deposition and collagen deposition in iron-overload mice. The expression of Bcl-2 was increased, the expression levels of Bax and caspase-3 were decreased under RES treatment. Moreover, RES reduced the expression of hepcidin, ferritin (Ft), divalent metal transporter-1 (DMT-1), transferrin receptor-2 (TFR-2), and raised the expression of ferroprotein-1 (FPN-1). In conclusion, RES could ameliorate iron overload-induced liver fibrosis, and the potential mechanisms may be related to antioxidant, anti-inflammatory, anti-apoptotic, and more importantly, regulation of iron homeostasis by reducing iron uptake and increasing iron export.

## Introduction

Iron is an essential microelement for humans and is involved in vital physiological procedures such as cell multiplication and differentiation, energy metabolism, and detoxification. However, excess iron can be highly toxic by catalyzing the production of reactive oxygen species (ROS) that destroy cells, tissues, and organs (Mehta, Farnaud & Sharp, 2019; Nairz & Weiss, 2006). Iron overload, induced by essential hemochromatosis and secondary iron overload (such as thalassemia and chronic virus hepatitis), is common worldwide (Krittayaphong et al., 2017). Excess iron is deposited in various tissues, such as the heart, kidneys, and endocrinal tissues, but the liver is the most common organ (Sikorska, Bernat & Wroblewska, 2016). Patients with iron overload disease may slowly develop hepatic fibrosis over an extended period of time eventually leading to cirrhosis, possibly liver carcinoma (Adams et al., 2006).

The liver participates in iron transport and regulates iron homeostasis. Proteins involved in hepatic iron transport or regulation have been identified, such as transferrin receptor (TFR) and divalent metal transporter 1 (DMT-1) (Graham et al., 2007). TFR-2 is a membrane-binding glycoprotein that can mediate cellular transferrin and iron uptake (Robb, Ericsson & Wessling-Resnick, 2004; Kawabata et al., 1999). Cells take up transferrin-dependent iron through TFR and store iron in the cytoplasm as ferritin (Ft) (Hentze et al., 2010). When iron overload, transferrin is supersaturated, and transferrin-independent iron (Fe^2+^) enters the liver through channels such as DMT-1 (Zhang et al., 2016). Excess Fe^2+^ in its free and insoluble form enters the organs and deposits in local tissues, causing cytomembranes and organelles damage (Brissot et al., 2012). Furthermore, studies have shown that ferroprotein (FPN) appears to be the sole mediator of iron release from hepatocytes, while hepcidin regulates iron homeostasis by binding FPN-1 extracellularly to internalize and degrade FPN-1 in lysosomes (Donovan et al., 2005; Nemeth et al., 2004).

Iron chelators such as deferiprone and deferoxamine (DFO) are currently utilized to treat iron-overloaded diseases (Heli, Mirtorabi & Karimian, 2011). However, long-term use of DFO leads to agranulocytosis, rash and renal failure (Maggio, 2007; Delea et al., 2007). Therefore, it is particularly important to explore new and effective drugs with few side effects for the treatment of iron-overload induced liver fibrosis.

Resveratrol (RES) is a non-flavonoid polyphenolic compound found in natural plants such as *cuspidatum*, *Vitis* and mulberry (Mirhadi et al., 2021). Previous studies indicated that RES has various bioactivities including antioxidant, anti-inflammatory, antifibrotic etc (Abdu & Al-Bogami, 2019; Jiang et al., 2016). Further, RES has been shown to directly protect the liver from iron-mediated influnce (Das et al., 2016). Previous studies have shown that RES can alleviate liver fibrosis caused by carbon tetrachloride or dimethylammonium nitrate by inhibiting lipid peroxidation. (Yu et al., 2019; El-Agamy, Shebl & Said, 2011).

On the basis of this information, this study hypothesized that RES protects against iron overload-induced liver fibrosis. First, this research explored the antifibrotic effects of RES by analyzing morphological and biochemical indicators and the expression of fibrosis-related proteins. To further analyze the potential mechanism of RES in protecting liver fibrosis caused by iron overload, this study analyzed the changes of oxidative stress indicators, inflammatory and apoptotic factors, and the expression of proteins related to iron homeostasis regulation. Despite studies illustrating the antifibrotic effect of RES, it remains unclear whether RES can achieve protective effects against iron overload-induced liver fibrosis by regulating iron homeostasis. Therefore, this study mainly explored the protective mechanisms of RES against iron overload-induced liver fibrosis.

## Materials & Methods

### Chemicals

**Figure 1:**
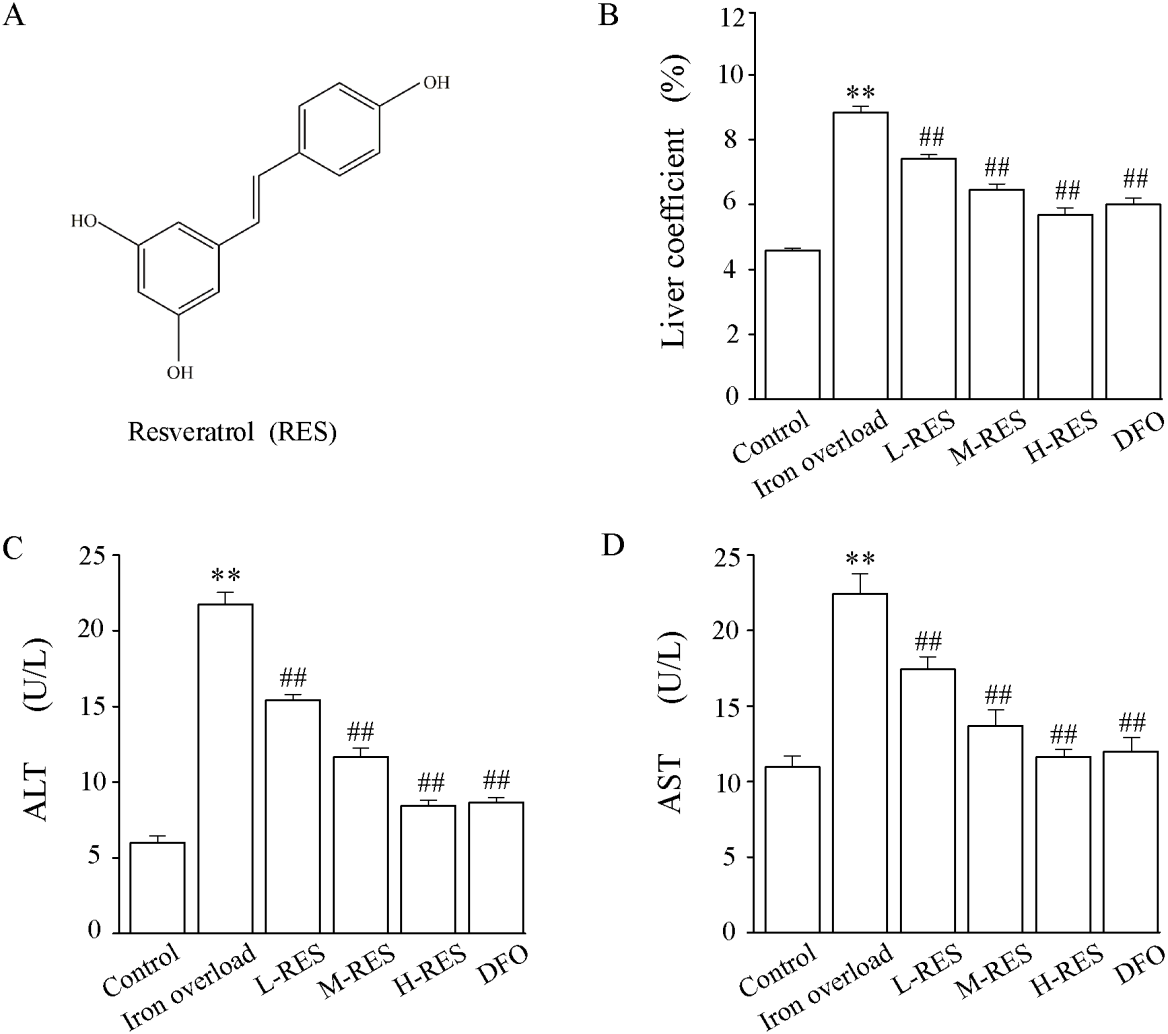
(A) The chemical structure of resveratrol (RES). (B) Effect of RES on liver coefficients in iron-overloaded mice. Data are expressed as the mean ± SEM (*n* = 10). (C) Effects of RES on activities of ALT and AST in iron-overloaded mice. Data are expressed as the mean ± SEM (*n* = 6). ^**^P < 0.01 *versus* control group, and ^##^*P* < 0.01 *versus* iron overload group.

RES (Fig. 1A) was obtained from Rhawn Reagent Co. (Shanghai, China). Sodium carboxymethylcellulose (purity 98%, 1,200 cps) was supplied by Behringer Technology Co., Ltd. (Beijing, China). DFO was obtained from Novartis Pharma AG (Basel, Switzerland). Iron dextran was supplied by Harbin Hongda Animal Medicine Factory (Harbin, China). The commercially available kits of alanine aminotransferase (ALT), aspartate aminotransferase (AST), hydroxyproline (Hyp), superoxide dismutase (SOD), malondialdehyde (MDA), glutathione (GSH), glutathione peroxidase (GSH-PX) and serum iron and liver iron were supplied by Jian Cheng Biological Engineering Institute (Nanjing, China).

### Animals

Sixty male Kunming mice (age, 6–7 weeks; 20.0 ± 2.0 g) were provided by Henan skbex Biotechnology Co., Ltd. Sixty mice were placed in twelve cages and the animals were raised in controlled conditions (22 ± 2 °C, 45∼55% relative humidity, 12-h day and night cycle) and fed food and water ad libitum during the experiment. This study has taken appropriate steps to minimize the suffering of the animals. All animal procedures were conducted in line with the Guidelines of Animal Experiments from the Committee of Medical Ethics, National Health Department of China. The experimental procedures were approved by the Ethics Committee for Animal Experiments of Hebei University of Chinese Medicine (DWLL2019040).

### Experimental protocol

After a week of adaptive feeding, 60 Kunming mice were stochastically assigned to one of six groups (*n* = 10 in per group): the control group, iron overload group, low dose RES group (L-RES, 25 mg/kg), medium dose RES group (M-RES, 50 mg/kg), high dose RES group (H-RES, 100 mg/kg) and DFO group (100 mg/kg). The doses of RES and DFO used were according to relevant studies (Zhang et al., 2019; Zhang et al., 2017; Zhang et al., 2013). Iron dextran (50 mg/kg) was injected intraperitoneally in all groups except the control group. Mice in the L-RES, M-RES and H-RES groups were gavaged with RES solution at a concentration 25, 50 and 100 mg/kg (the sodium carboxymethyl cellulose and ionized water were fused in a ratio of 1:200 for the preparation RES solution), respectively, 4 h before the daily injection of iron dextran; mice in the DFO group were injected with DFO intraperitoneally; and the control group mice received isovolumetric saline. The duration of the experiment was 7 weeks. All mice were anesthetized using sodium pentobarbital (50 mg/kg) 24 h after the last iron dextran administration, and blood was collected by enucleating the mice eyeball. The centrifuged serum was used for further analysis. The liver was rapidly removed, frozen in liquid nitrogen, or fixed in 4% buffered formalin for further analysis. Sample size calculation was based on previous experience as well as experimental requirements to determine significant differences.

### Assessment of biochemical indicators

The activities of serum ALT (Catalog: C009-2-1) and AST (Catalog: C010-2-1), and the activities of serum GSH (Catalog: A006-2-1) were evaluated using commercially available kits based on microplate method. Determination of GSH-PX (Catalog: A005-1-2) activities were performed using commercially available kits based on the colorimetric method. Determination of SOD (Catalog: A001-3-1) and MDA (Catalog: A003-1-1) levels were performed based on WST-1 and thiobarbituric acid method, respectively. (Jian Cheng Biological Engineering Institute, Nanjing, China) (Zhang et al., 2020).

### Morphological analyses

At the end of the experiment, the body and liver weights of the mice in each group were collected, and the liver weight coefficient was calculated as the percentage of liver weight and body weight. Liver samples were fixed with 4% paraformaldehyde, embedded in paraffin and cut into 4-µm-thick sections. The samples were stained with hematoxylin-eosin (H&E), Prussian blue and Masson according to the manufacturer’s procedure. The pathological changes in liver of each group were visualized by optical microscopy (Leica DM4000B, Solms, Germany). Nearly all areas of the three sections per group were observed, and representative images were selected for acquisition. Images were semiquantitatively analyzed using ImageJ software.

### Assessment of liver iron and serum iron content

Determination of liver iron (Catalog: A039-2-1) and serum iron (Catalog: A039-1-1) contents were performed using commercially available kits based on the colorimetric method according to the brochures of manufacturers (Jian Cheng Biological Engineering Institute, Nanjing, China).

### Measurement of liver Hyp content

Hyp is a collagen-specific amino acid that is used to measure collagen. In this study, Hyp content was estimated using colorimetric determination on the basis of the reaction of oxygenated Hyp with p-dimethylaminobenzaldehyde. Hydrolyzed 100 mg wet hepatic tissue was placed in one mL of the solution at 95 °C for 20 min and diluted to 10 mL with distilled water followed by centrifugation (3,500 rpm, 10 min) to acquire one mL supernatant. Finally, the absorbance value was detected spectrophotometrically at a wavelength of 550 nm (Zhang et al., 2013).

### Inflammatory factor measurement by ELISA

Liver samples from different groups of mice were collected and frozen in liquid nitrogen for the determination of inflammatory factors. The levels of tumor necrosis factor-α (TNF-α, Catalog: CSB-E04640r) and interleukin-6 (IL-6, Catalog: CSB-E11987r) in liver tissues were detected by ELISA kits. According to the manufacturer’s instructions, tissue supernatant, enzyme labeling reagent, and chromogenic reagent were added to each well, after incubation, washing, and stop solution was added, and then the OD value of each well was measured at 450 nm using a microplate reader. (SenBeiJia Biological Technology Co., Ltd. Nanjing, China) (Sun et al., 2021).

### Western blotting

The liver tissue was homogenized by RIPA buffer (BeiJing Solarbio Technology Co., Ltd) and centrifuged at 12,000 rpm for 10 min at 4 °C. The supernatant was collected and stored in aliquots at –80 °C. The protein was isolated by sodium dodecyl sulfate-polyacrylamide gel electrophoresis (SDS-PAGE) and transferred to PVDF membranes. The membranes were blocked with blocking solution containing 5% nonfat milk for 2 h at room temperature, and the membranes were incubated with the following primary antibodies at 4 °C overnight: anti-caspase-3 (Catalog: 66470-2-Ig, 1:1500; Proteintech, Wuhan, China), anti-Bcl-2 (Catalog: 26593-1-Ig, 1:1200; Proteintech, Wuhan, China), anti-Bax and anti-type I collagen (Col-I) (Catalog: 60267-1-Ig and Catalog: 14695-1-AP, 1:1200; Proteintech, Wuhan, China), anti-α-smooth muscle actin (Catalog: 14395-1-AP, 1:1500; Proteintech, Wuhan, China), anti-hepcidin (Catalog: PAB979Mu01, 1:1000; Cloud-clone, Wuhan, China), anti-Ft (Catalog: ab75973, 1:1500; Abcam, Shanghai, China), anti-DMT-1 (Catalog: 20507-1-AP, 1:800; Proteintech, Wuhan, China) anti-TFR-2 and anti-FPN1 (Catalog: PAA262Ra01 and Catalog: PAC489Mi01, 1:800; Cloud-clone, Wuhan, China) and antibody β-actin (Catalog: TA-09, 1:1200; Zhong Shan Golden Bridge, Beijing, China). Next, the membranes were washed three times with PBS at room temperature for 10 min each. The membranes were incubated with secondary antibodies (1:3000) for 90 min and washed three times with PBS. Proteins were visualized by ECL detection reagent (Catalog: sc-2048; Zhong Shan Golden Bridge, Beijing, China) and X-ray film. Tanon Gis software was used to detect the gray value of ferritin, after which the results were analyzed.

### Statistical analysis

Values were presented as mean ± standard error of the mean (SEM). Data were statistically analyzed by one-way analysis of variance (ANOVA) and Tukey’s test using Origin Pro version 9.1 software. *P* < 0.01 or *P* < 0.05 was considered statistically significant.

## Results

### Effect of RES on liver weight coefficients

Compared with the control group, the liver weight coefficient of mice in the iron overload group was significantly increased by 1.92-fold. In contrast to the iron overload group, the liver weight coefficient was decreased by 16.26% in the L-RES group, 27.03% in the M-RES group, and 35.58% in the H-RES group (Fig. 1B). These suggests that RES can reduce the increase in liver weight coefficient caused by iron overload in a dose-dependent manner.

### Effect of RES on ALT and AST levels

Changes in liver function were estimated by the activities of ALT and AST (Figs. 1C and 1D). Relative to controls, the ALT (by 3.62-fold) and AST (by 2.04-fold) values of mice in the iron overload group were dramatically elevated (*P* < 0.01). Relative to the iron overload group, the DFO (ALT: −60%; AST: −46.5%) group and L-RES (ALT: −29.07%; AST: −22.3%), M-RES (ALT: −46.27%; AST: −39.03%), H-RES (ALT: −61.18%; AST: −48.13%) groups had markedly reduced the ALT and AST levels (*P* < 0.01).

### Morphological changes

#### Liver injury visualized by H&E staining

**Figure 2:**
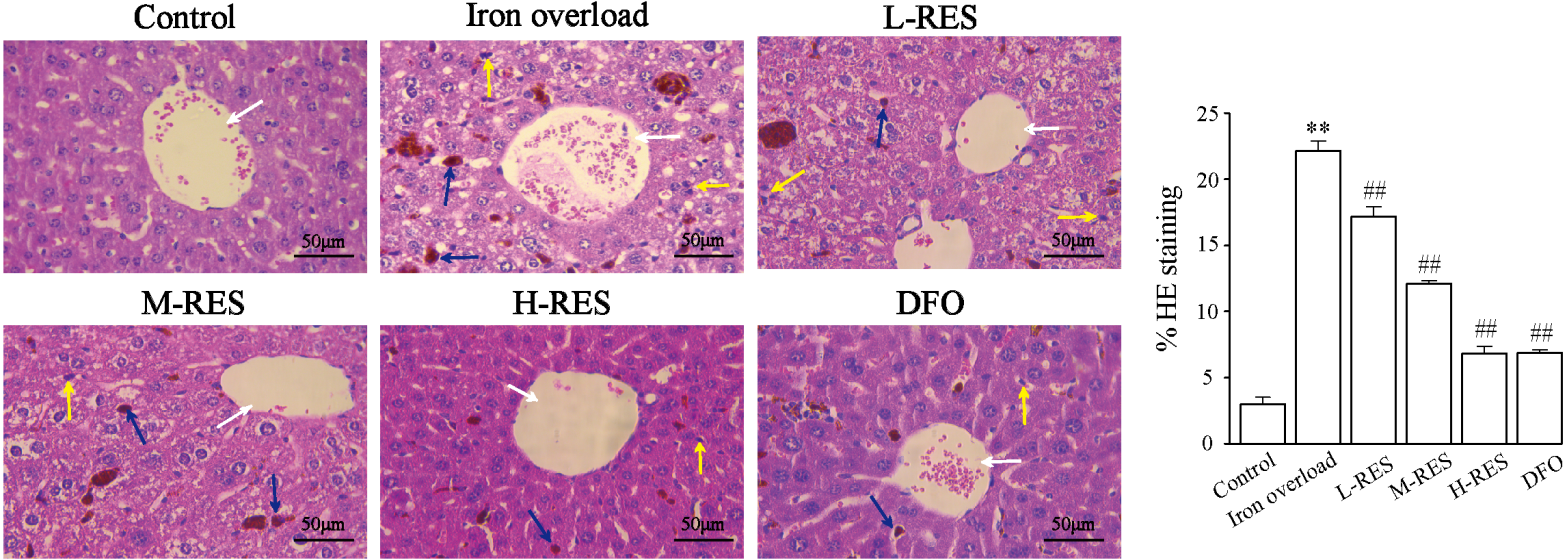
Effects of RES on pathologic changes of liver were visualized by H&E staining. Scale bar, 50 µm; magnification, ×400. The control group showed normal structure, inflammatory cell infiltration and yellow brown iron deposits can be seen from the iron overload group, while L-RES, M-RES, H-RES and DFO groups improved hepatic morphological changes. Central vein (white arrows), lymphocytes (yellow arrows) and Kupffer cells (blue arrows) were represented in H&E sections. ^**^P < 0.01 *versus* control group, ^##^*P* < 0.01 *versus* iron overload group.

Under a light microscope, observations of liver sections by H&E staining illustrated that hepatocytes were in a radially centered arrangement in the central vein, and hepatic lobule’s structure was clear in the control group (Fig. 2). The iron-overload liver tissue showed many yellowish-brown deposited plates, and inflammatory cell infiltration in the liver interstitium, and the hepatocytes were arranged in a disordered manner. After administration of DFO, L-RES, M-RES, and H-RES, the degree of liver injury was significantly reduced, inflammatory cell infiltration was decreased, and the arrangement of hepatocytes was relatively neat.

#### Iron deposition was visualized by Prussian blue staining

**Figure 3:**
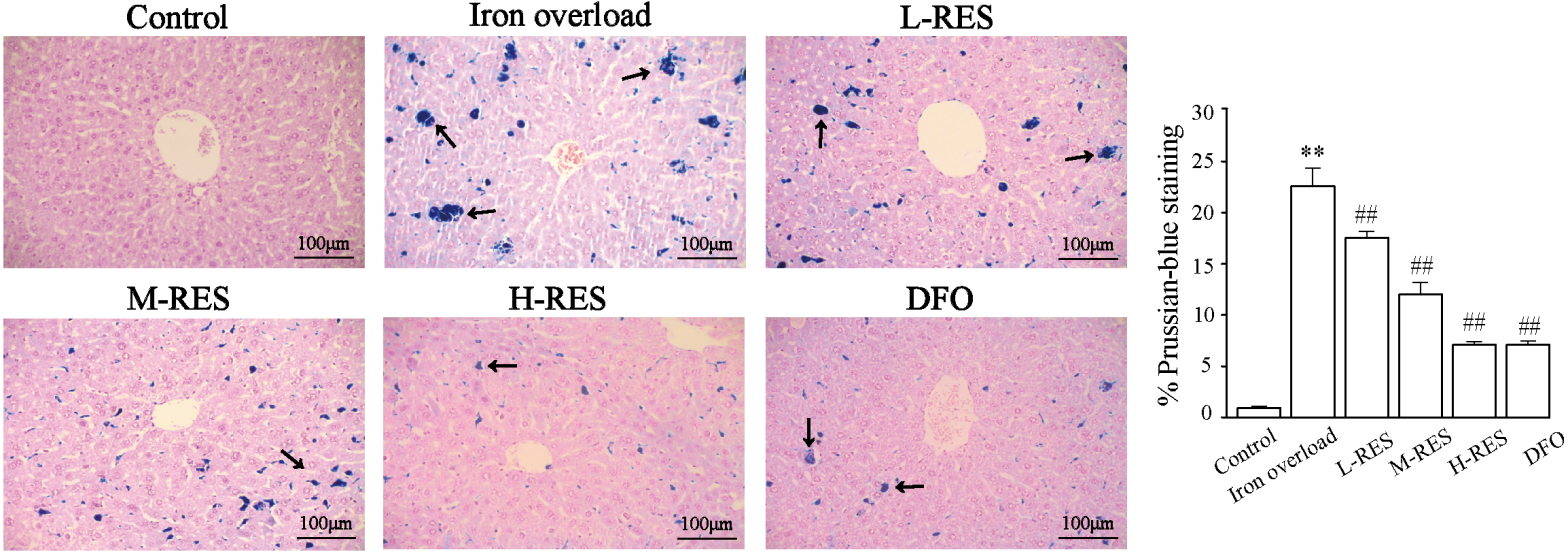
Effects of RES on liver iron deposition were visualized by Prussian blue staining. Scale bar, 100 µm; magnification, ×200. The abnormal distribution of iron deposits is illustrated by blue pigmentation. ^**^P < 0.01 *versus* control group, ^##^*P* < 0.01 *versus* iron overload group.

Prussian blue staining was employed to detect iron deposition in liver. Blue iron deposition was not observed in the control group (Fig. 3), but a large blue iron deposition could be found in the iron overload group. Upon administration of RES, iron deposition was reduced in a concentration-dependent manner. Hepatic iron deposition was also significantly reduced in the DFO group.

### Effect of RES on liver iron and serum iron content

**Figure 4:**
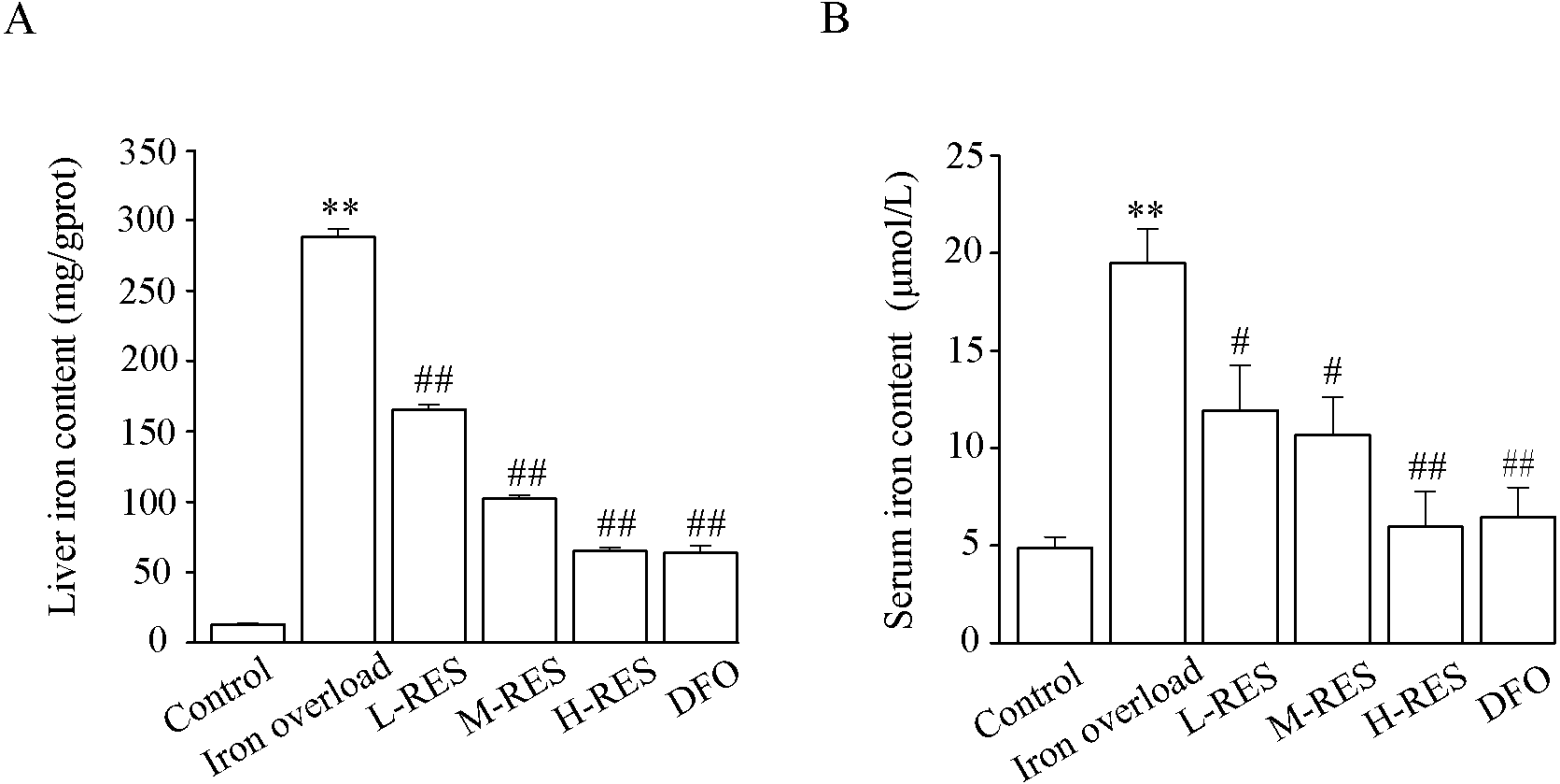
Effects of RES on liver iron (A) and serum iron (B) contents of iron-overloaded mice. Data are presented as the mean ± SEM (*n* = 6). ^**^P < 0.01 *versus* control group, ^#^*P* < 0.05 and ^##^*P* < 0.01 *versus* iron overload group.

Figure 4 shows the change in liver and serum iron content. Compared to controls, liver iron (by 4-fold) and serum iron (by 22.04-fold) contents were obviously increased in the iron overload group (*P* < 0.01). Upon administration of RES, liver iron (−39%, −45%, −69%) and serum iron (−42.53%, −64.4%, −77.45%) contents were reduced in a concentration-dependent manner, as did liver iron (−77.81%) and serum iron (−67%) contents in the DFO group (*P* < 0.05 or *P* < 0.01).

### Effect of RES on oxidative stress

#### Effect of RES on serum SOD, MDA, GSH activities

**Figure 5:**
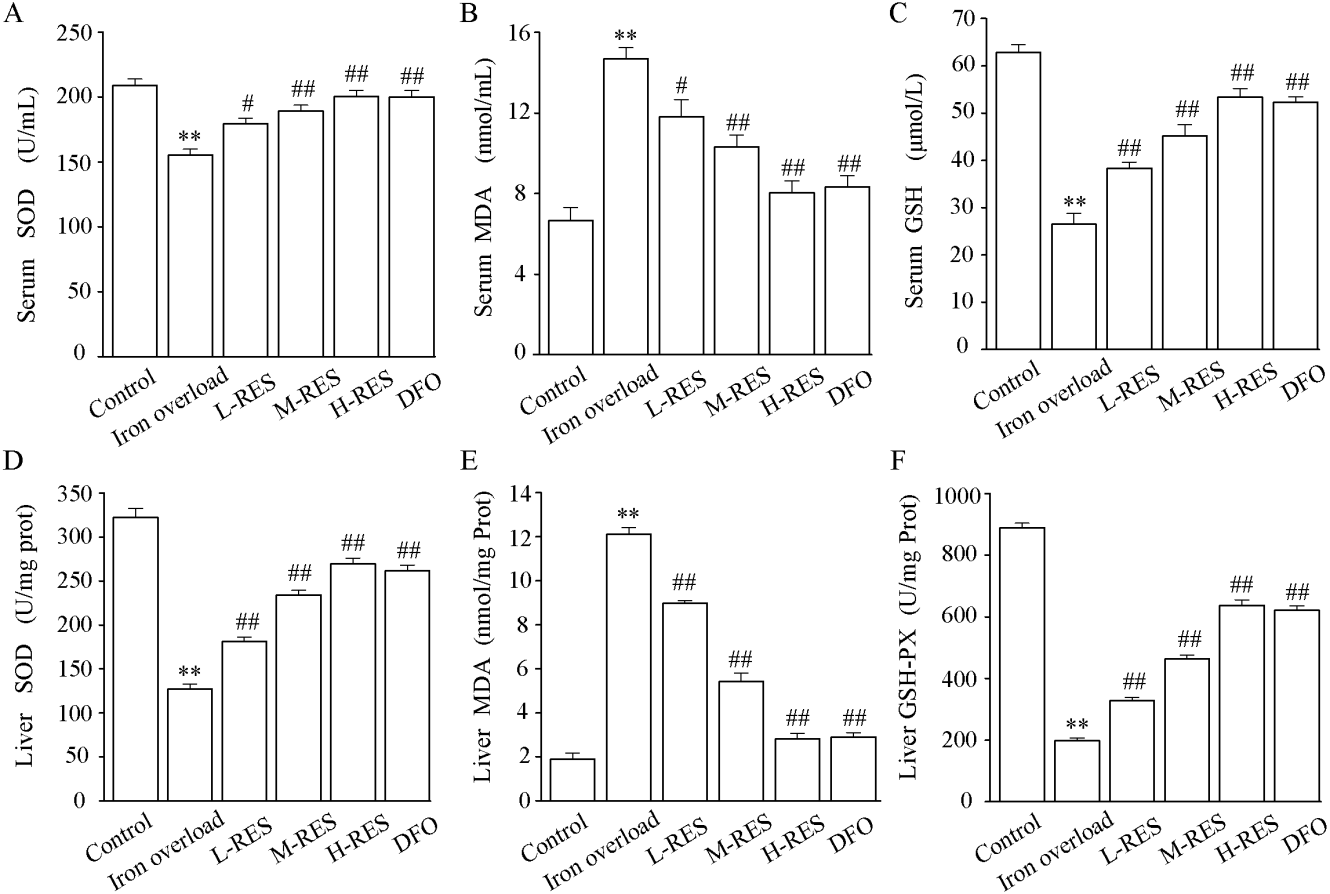
Effects of RES on SOD (A), MDA (B) GSH (C) and in the serum of iron-overloaded mice. Effects of RES on liver SOD (D), MDA (E) GSH-PX (F) in iron-overloaded mice. Values are presented as the mean ± SEM (*n* = 6). ^**^P < 0.01 *versus* control group, ^#^*P* < 0.05 and ^##^*P* < 0.01 *versus* iron overload group.

The activities of SOD and GSH and the content of MDA in serum are shown in Figs. 5A–5C. In contrast to controls, SOD (−25.71%) and GSH (−57.89%) levels were significantly decreased in the iron overload group (*P* < 0.01), whereas MDA (by 2.2-fold) was markedly increased (*P* < 0.01). Compared with the iron overload group, SOD (+15.58%, +21.77%, +28.94%) and GSH (+44.88%, +70.87%, +101.57%) levels in the L-RES, M-RES, and H-RES groups increased in a concentration-dependent manner, whereas MDA (−19.59%, −29.9%, −45.36%) was reduced (*P* < 0.05 or *P* < 0.01).

#### Effect of RES on liver SOD, MDA, GSH-PX activities

As shown in Figs. 5D–5F, compared with the control group, the liver SOD (−60.51%) and GSH-PX (−77.73%) activities were significantly decreased in the iron overload group mice, whereas MDA (by 6.42-fold) levels were significantly increased. In the L-RES, M-RES, and H-RES groups, the liver SOD (+29.83%, +83.95%, +112.16%) and GSH-PX (+65.61%, +134.35%, +221.96%) activities were significantly increased, and the MDA (−25.95%, −55.18%, −76.76%) levels were markedly decreased (*P* < 0.01).

### Effect of RES on inflammation

**Figure 6:**
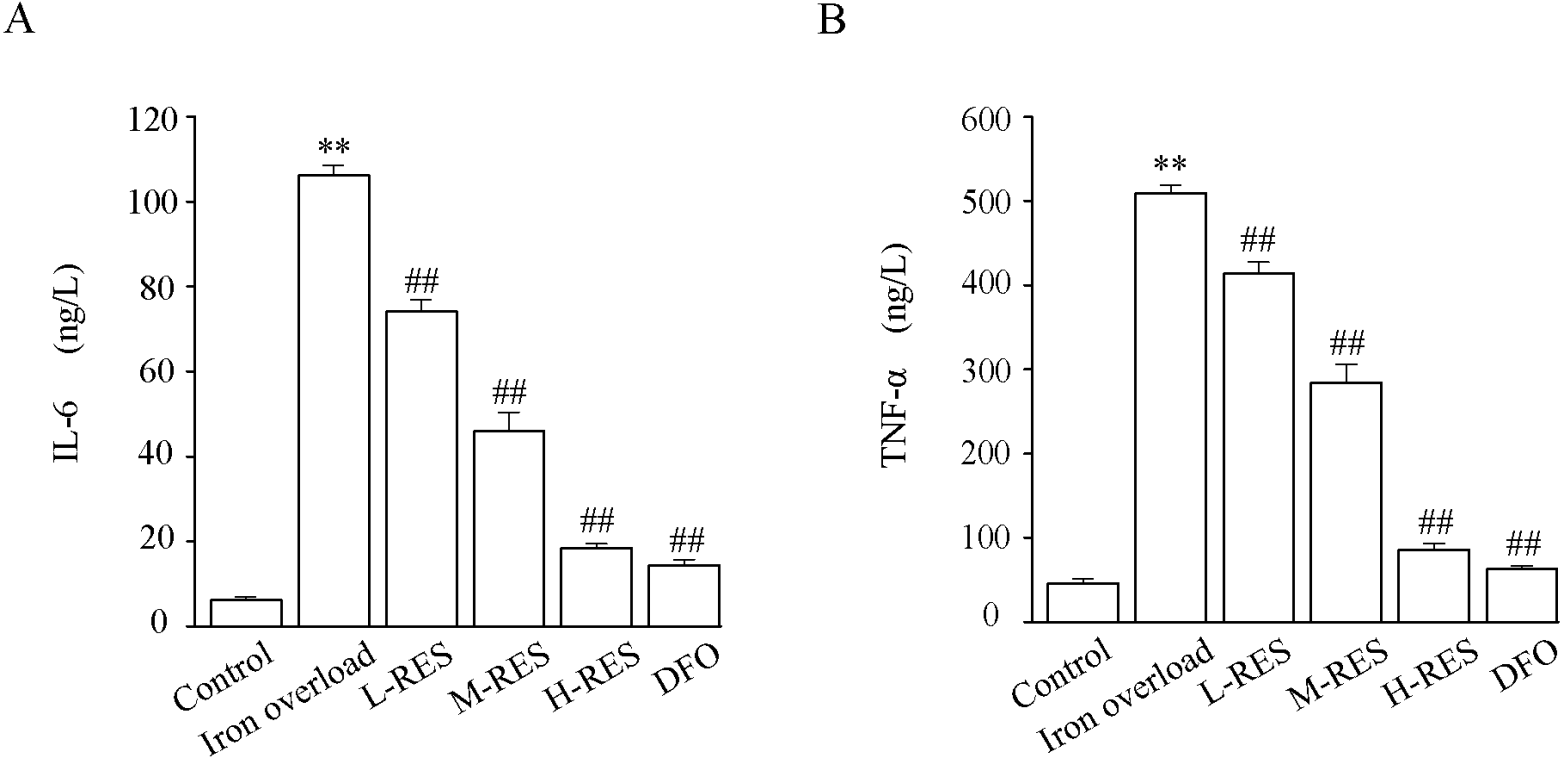
Effects of RES on IL-6 (A) and TNF-α (B) in liver of iron-overload mice. Values are presented as the mean ±SEM (*n* = 6). ^**^P < 0.01 *versus* control group, ^##^*P* < 0.01 *versus* iron overload group.

As Fig. 6 shown, the levels of TNF-α (by 11.1-fold) and IL-6 (by 17.2-fold) in the iron overload group were clearly higher than those in the control group (*P* < 0.01). Further, the levels of TNF-α (−18.68%, −44.18%, −83.18%; DFO: −87.54%) and IL-6 (−30.2%, −56.72%, −82.65%; DFO: −86.45%) were decreased in the RES and DFO groups (*P* < 0.01).

### Effect of RES on apoptosis

**Figure 7:**
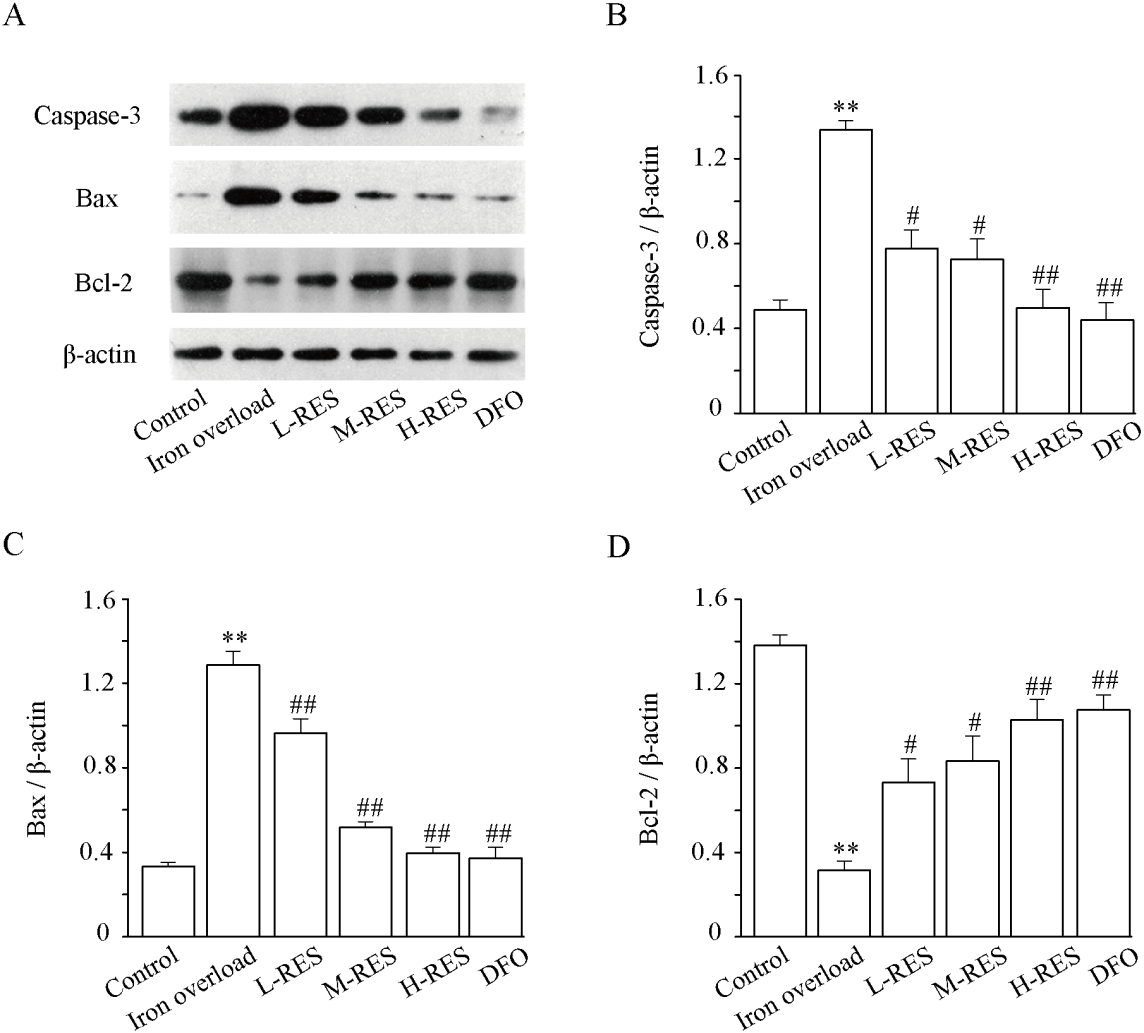
Effects of RES on caspase-3 (B), Bax (C) and Bcl-2 (D) in liver of iron-overload mice. Data are expressed as the mean ± SEM (*n* = 3). ^**^P < 0.01 *versus* control group, ^#^*P* < 0.05 and ^##^*P* < 0.01 *versus* iron overload group.

Expression levels of apoptosis-related proteins such as caspase-3, Bax and Bcl-2 were measured using Western blotting. (Fig. 7A). As illustrated in Fig. 7, relative to controls, the expression levels of Bax (by 3.84-fold) and caspase-3 (by 2.73-fold) in the iron overload group increased significantly (*P* < 0.01; Figs. 7B and 7C), whereas the expression of Bcl-2 (−77.21%) decreased (*P* < 0.01; Fig. 7D). In the RES and DFO groups, Bax (−25.14%, −29.58%, −69.16%; DFO: −70.96%) and caspase-3 (−41.9%, −45.75%, −62.77%; DFO: −66.91%) were downregulated, and Bcl-2 (+132.24%, +165.15%, +227.11% DFO: +241.93%) was upregulated (*P* < 0.05 or *P* < 0.01).

### Effect of RES on liver fibrosis

#### Hepatic fibrosis visualized by Masson staining

**Figure 8:**
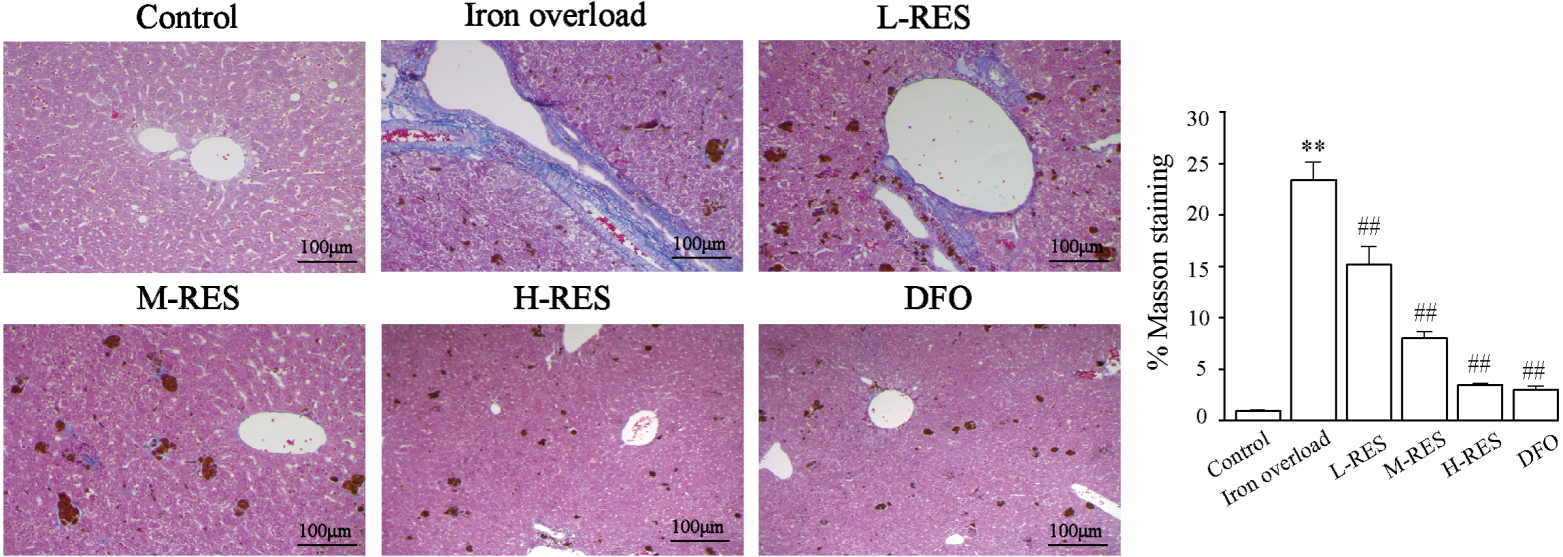
Effects of RES on the degree of liver fibrosis were observed by Masson staining. Scale bar, 100 µm; magnification, ×200. Blue matter indicates collagen fibrils.

Masson staining was used to observe the localization of collagen fibers in the hepatic structure. More collagen fibers stained light blue are seen in the iron overload group (Fig. 8). After RES and DFO treatment, the range of fibrosis was reduced in contrast to the iron overload group, showing that RES is capable of reducing liver fibrosis.

#### Effect of RES on α-SMA and COI-1 expression levels

**Figure 9:**
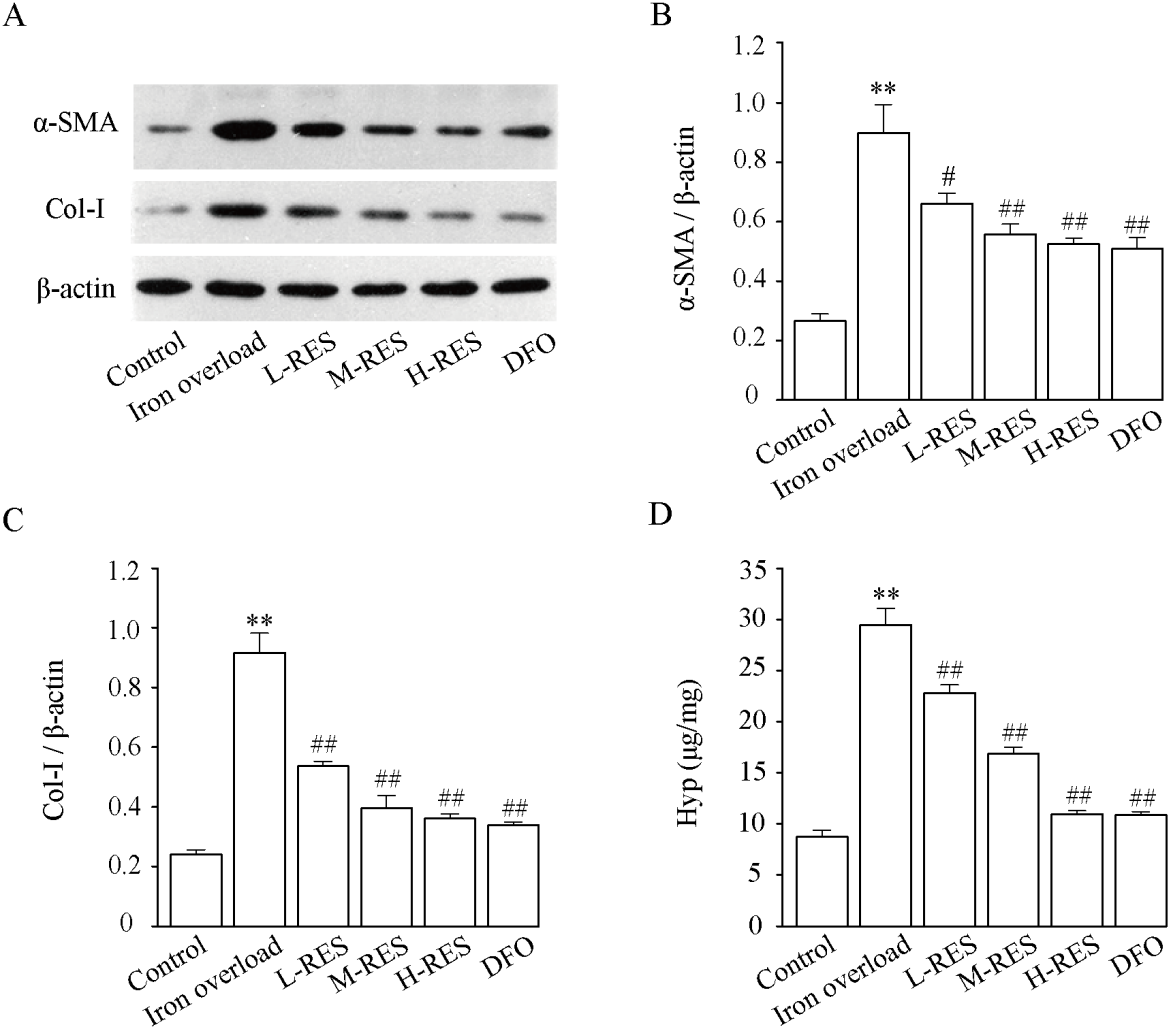
Effects of RES on α-SMA (B) and Col-I (C) in liver of iron-overload mice (*n* = 3). Effects of RES on liver Hyp (D) in mice with iron overloaded (*n* = 6). Data are presented as the mean ± SEM. ^**^P < 0.01 *versus* control group, ^#^*P* < 0.05 and ^##^*P* < 0.01 *versus* iron overload group.

Figure 9 shown that relative to controls, the expression of α-SMA (+238.7%, Fig. 9B) and Col-I (+281.13%, Fig. 9C) were dramatically increased in the iron overload group (*P* < 0.01). Compared with the iron overload group, the expression of α-SMA (−26.47%, −37.97%, −41.68%) and Col-I (−41.4%, −56.78%, −60.58%) decreased in a concentration gradient after taking L-RES, M-RES, and H-RES (*P* < 0.05 or *P* < 0.01).

#### Effect of RES on liver Hyp content

Hyp, a specific amino acid in collagen, is broadly used as an indicator to quantify the degree of fibrosis. Fig. 9D shows that relative to controls, the Hyp (+236.74%) content of liver in the iron overload group was clearly elevated (*P* < 0.01). These results indicated the presence of a large amount of collagen deposition in the liver of mice. In contrast to the iron overload group, Hyp (−22.65%, −42.78%, −62.84%; DFO: −63.1%) was significantly decreased in the liver after RES and DFO treatment (*P* < 0.01).

### Effect of RES on DMT-1, TfR-2, FPN-1, and Ft expression levels

**Figure 10:**
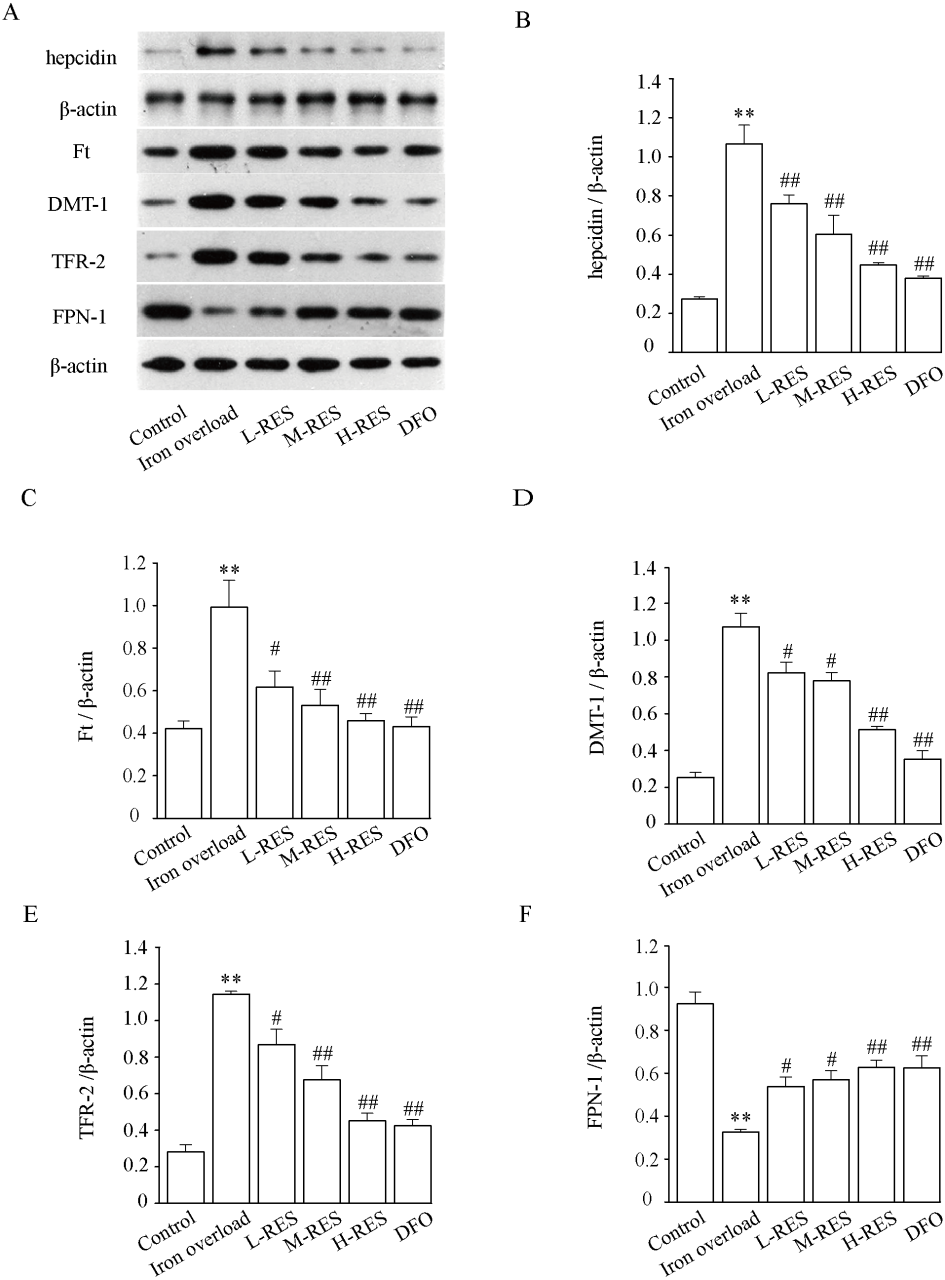
Effects of RES on hepcidin (B), Ft (C), DMT-1 (D), TFR-2 (E) and FPN-1 (F) in iron-overload mice. Data are expressed as the mean ± SEM (*n* = 3). ^**^P < 0.01 *versus* control group, ^#^*P* < 0.05 and ^##^*P* < 0.01 *versus* iron overload group.

Figure 10 demonstrates that hepcidin (+296.30%) and Ft (+134.93%) protein expression levels were obviously raised in the iron overloaded group relative to controls (*P* < 0.01). Whereas hepcidin (−28.97%, −43.93%, −58.88%; DFO: −64.49%) and Ft (−37.81%, −46.48%, −53.73%; DFO: −56.6%) protein expression levels were apparently decreased in RES and DFO groups, which indicates that RES can reduce the expression of hepcidin and Ft (*P* < 0.05 or *P* < 0.01). Under iron overload conditions, the expression levels of DMT-1 (+322.47%) and TFR-2 (+306.75%), which are related to hepatic iron transport, were upregulated, while FPN-1 (−64.76%) was down regulated (*P* < 0.01; Fig. 10). After RES and DFO treatment, the expression of TFR-2 (−24.1%, −40.87%, −60.49%; DFO: −62.95%) and DMT-1 (−23.26%, −27.34%, −52.23%; DFO: −67.06%) were down regulated, while FPN-1 (+65.33%, +75.38%, +92.6%; DFO: +91.92%) was upregulated (*P* < 0.05 or *P* < 0.01).

## Discussion

A literature review provides ample evidence for the important role of iron accumulation in the progression of liver fibrosis (Diwakaran et al., 2002). Excess iron promotes a Fenton reaction and produces free radicals, leading to cellular and tissue damage, promoting the development of liver fibrosis (Mehta, Farnaud & Sharp, 2019). RES is a polyphenolic compound with various pharmacological effects: antioxidant, anti-inflammatory, and anti-apoptotic, among others (Chávez et al., 2008). In multiple models, RES has been shown to ameliorate liver injury and fibrosis (Che et al., 2020; Cheng et al., 2019; Zhu et al., 2020). In addition, RES protects against liver injury from iron overload by reducing iron deposition (Li et al., 2021). However, the underlying mechanism of RES ameliorates iron overload-induced liver fibrosis remains unclear.

In this study, a mouse model of iron overload was established by intraperitoneal injection of iron dextran. This study shows that serum ALT and AST levels increased in iron overloaded mice (Figs. 1C–1D); the morphology indicates inflammatory cell infiltration (Fig. 2), iron deposition (Fig. 3), and an increased area of fibrosis in the liver (Fig. 8). This is consistent with a previous study that uses iron overload-induced liver fibrosis in a model (Zhang et al., 2013).

Excessive iron deposition in the liver will promote oxidative stress, release oxygen free radicals, cause lipid peroxidation, further aggravate liver injury, and promote the occurrence and development of liver fibrosis (Mehta, Farnaud & Sharp, 2019; Galaris & Pantopoulos, 2008). A large amount of ROS is generated by excess iron in the liver. An excessive accumulation of ROS creates an abnormal inflammatory response for hepatocyte apoptosis or necrosis in the cells (Ramm & Ruddell, 2005). This study present evidence for RES-inhibited oxidative stress by increasing SOD and GSH activities and by decreasing MDA levels (Fig. 5), meanwhile, it improved inflammation and hepatocyte apoptosis. This is consistent with studies showing how RES ameliorates liver fibrosis by inhibiting oxidative stress (Hessin et al., 2017; Yu et al., 2019).

After injection of iron dextran, Prussian blue shows excessive iron deposition in the liver (Fig. 3), resulting in increased hepatic iron levels (Fig. 4), hepatocyte damage and apoptosis, and promoting the development of liver fibrosis. In addition, elevated serum iron levels may be due to liver cell damage (Malik et al., 2017). In this study, after RES treatment, liver iron levels were decreased by reducing hepatic iron deposition, resulting in amelioration of hepatocellular injury. Therefore, reducing hepatic iron deposition may be a vital mechanism for RES to ameliorates iron overload-induced liver fibrosis.

The liver is the main storage site for iron, and the central regulator of iron homeostasis in the body (Rishi & Subramaniam, 2017; Bloomer & Brown, 2019). Liver hepcidin is considered a major contributor to the systemic regulation of iron, while the liver also maintains systemic iron homeostasis by regulating hepcidin levels. In the presence of excess iron, TFR become saturated with iron imported into the liver *via* the DMT-1 channel in the form of Fe^2+^ by TFR-mediated endocytosis. Given excess iron deposits in the liver, cells take up transferrin-dependent iron through TFR, and store it in the cytoplasm as Ft, causing hepatocyte damage and the development of liver fibrosis (Brissot et al., 2012; Iron, 2005). Iron absorption and tissue distribution are mainly controlled by the interaction of hepatic hepcidin with FPN. Excess iron allows hepatocytes to produce more hepcidin, which regulates iron homeostasis by binding FPN-1 extracellularly to internalize and degrade FPN-1 in lysosomes (Nemeth & Ganz, 2021). This study found that expression levels of hepcidin, Ft, channel proteins DMT-1 and TFR-2 increased in the iron overload group, while expression levels of FPN-1 decreased. However, RES downregulated expression levels of hepcidin, Ft, channel proteins DMT-1 and TFR-2, and up-regulated the expression levels of FPN-1 protein (Fig. 10). These suggests that reduced iron uptake and increased iron export may be underlying mechanisms by which RES regulates iron homeostasis.

**Figure 11:**
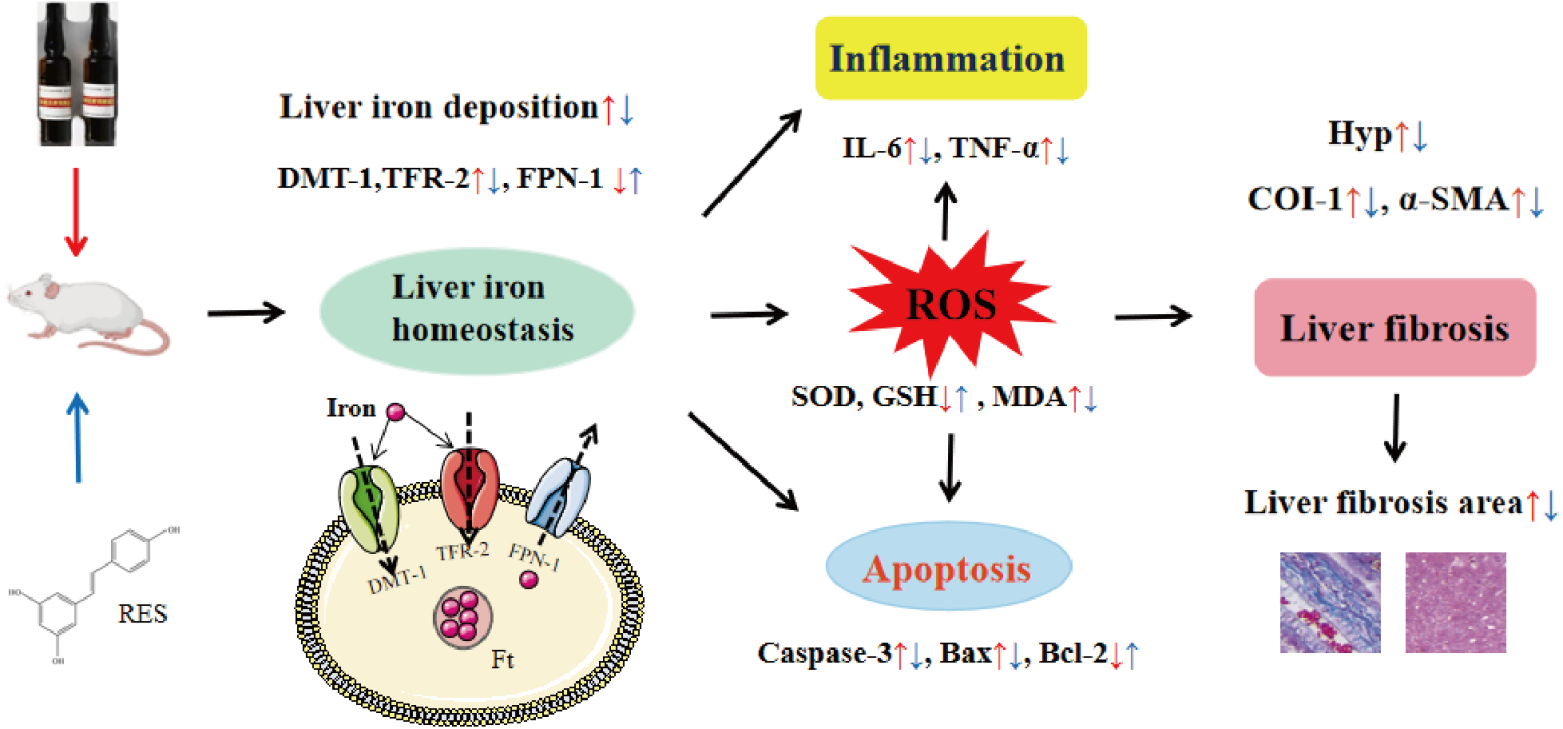
Diagrammatic sketch for the protective effects of RES against iron overload-induced liver fibrosis.

In conclusion, this study shows that RES ameliorates iron overload-induced liver fibrosis. The potential mechanisms may be related to antioxidant, anti-inflammatory, anti-apoptotic, and more importantly, regulation of iron homeostasis by inhibiting the expression of iron uptake-related proteins DMT-1 and TFR-2 and promoting the expression of iron excretion protein FPN-1 (Fig. 11). This research provides an experimental basis for RES as a treatment agent for liver fibrosis in iron overload diseases. However, there is still much work to be done for the convincing clinical efficacy of RES in the treatment of liver fibrosis caused by iron overload.

## Supplemental Information

10.7717/peerj.13592/supp-1 Supplemental Information 1 Raw data

10.7717/peerj.13592/supp-2 Supplemental Information 2 Author Checklist

## Additional Information and Declarations

### Data Availability

The following information was supplied regarding data availability: All raw data are available in the Supplemental File.
