# Supplementary figures and images for: Resveratrol ameliorates iron overload induced liver fibrosis in mice by regulating iron homeostasis
Source: PeerJ. 2022 Jun 8;10:e13592. doi: 10.7717/peerj.13592 (PMC9188311; doi:10.7717/peerj.13592)

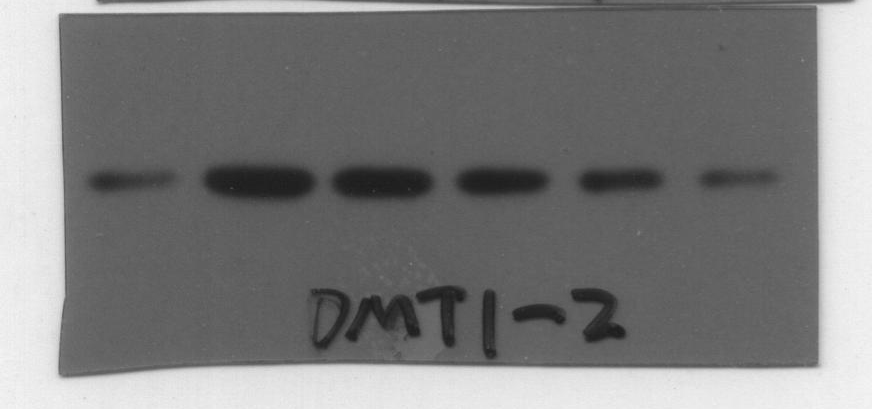

Supplement: Supplemental Information 1 [file peerj-10-13592-s001.zip › Raw data/Fig.10/DMT1 (2).jpg]

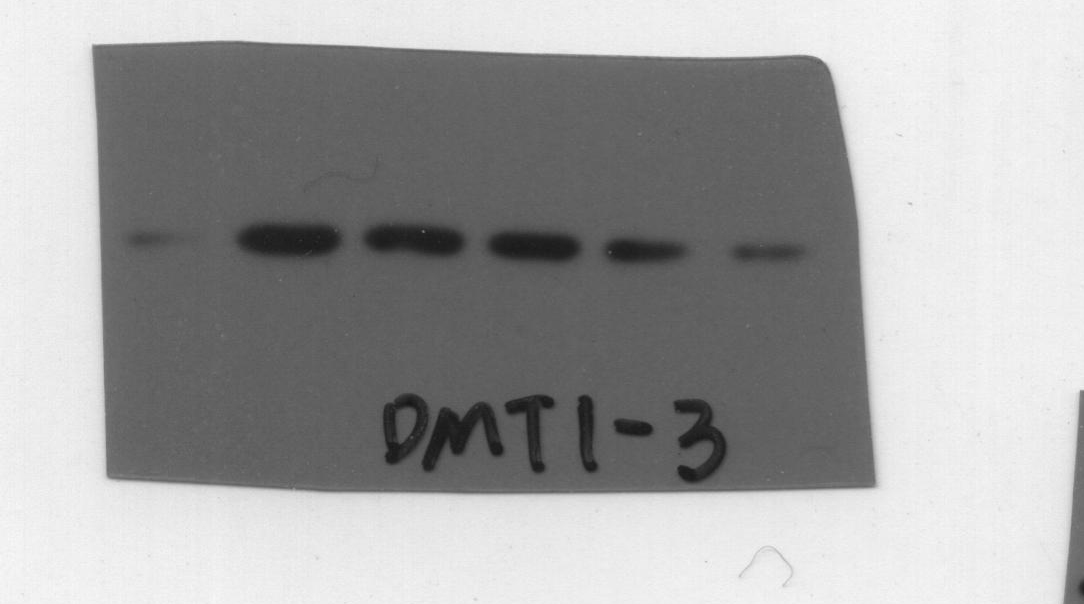

Supplement: Supplemental Information 1 [file peerj-10-13592-s001.zip › Raw data/Fig.10/DMT1 (3ú⌐.jpg]

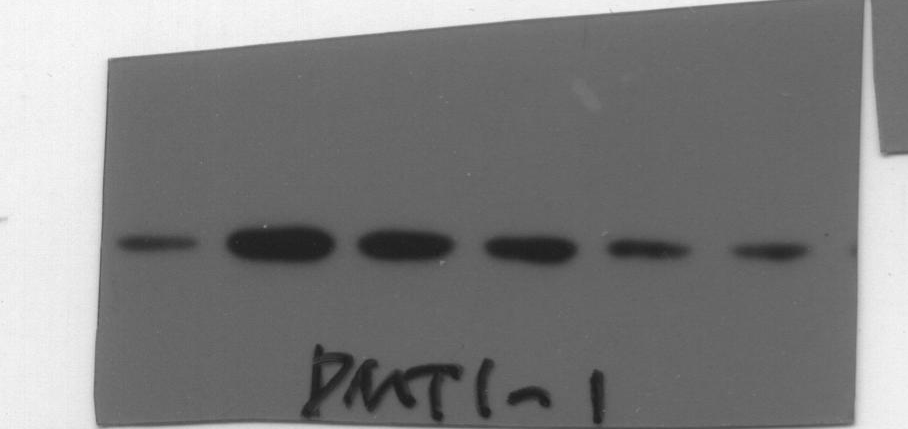

Supplement: Supplemental Information 1 [file peerj-10-13592-s001.zip › Raw data/Fig.10/DMT1 ú¿1ú⌐.jpg]

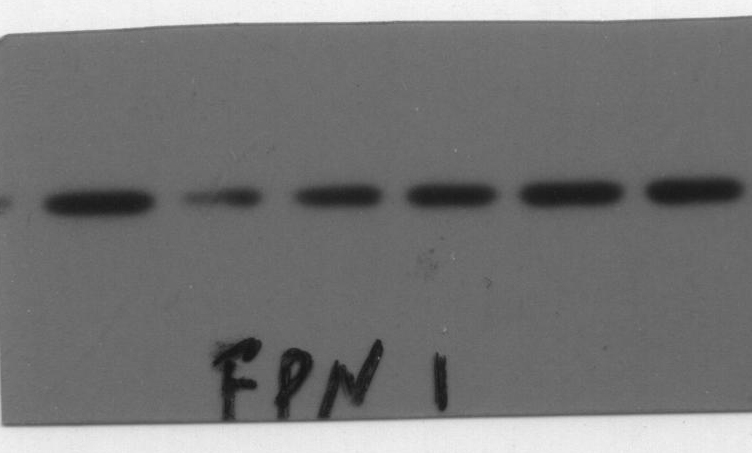

Supplement: Supplemental Information 1 [file peerj-10-13592-s001.zip › Raw data/Fig.10/FPN-1 ú¿1ú⌐.jpg]

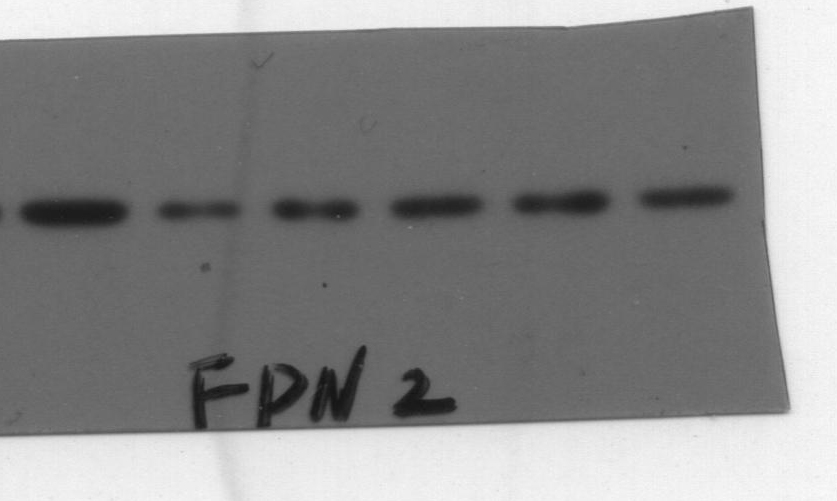

Supplement: Supplemental Information 1 [file peerj-10-13592-s001.zip › Raw data/Fig.10/FPN-1 ú¿2ú⌐.jpg]

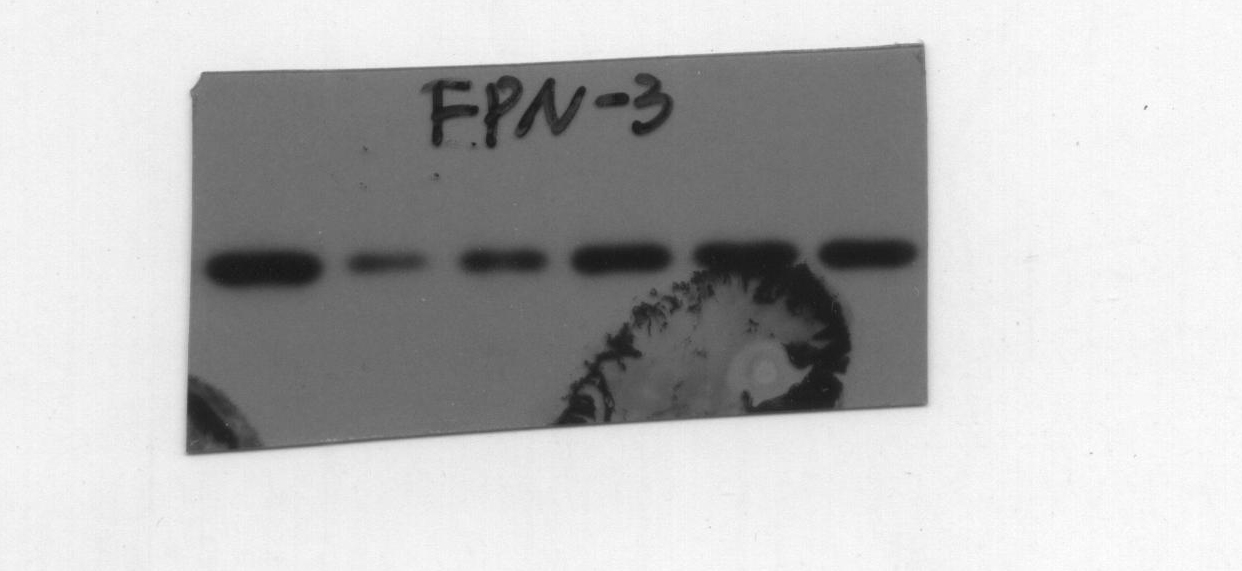

Supplement: Supplemental Information 1 [file peerj-10-13592-s001.zip › Raw data/Fig.10/FPN-1 ú¿3ú⌐.jpg]

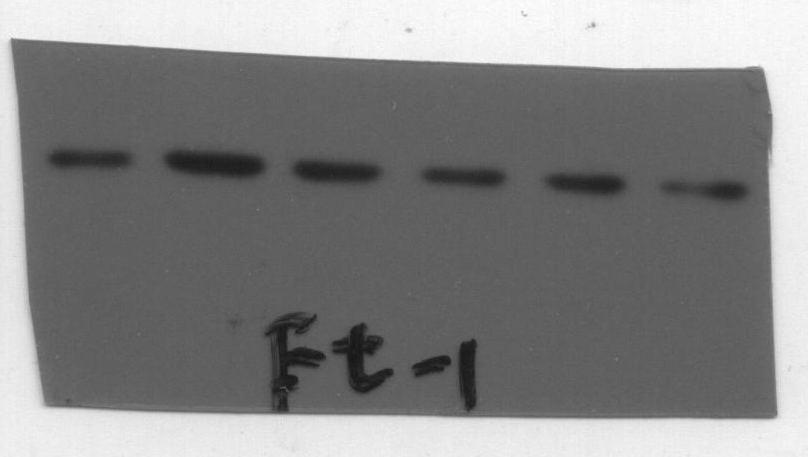

Supplement: Supplemental Information 1 [file peerj-10-13592-s001.zip › Raw data/Fig.10/Ft (1).jpg]

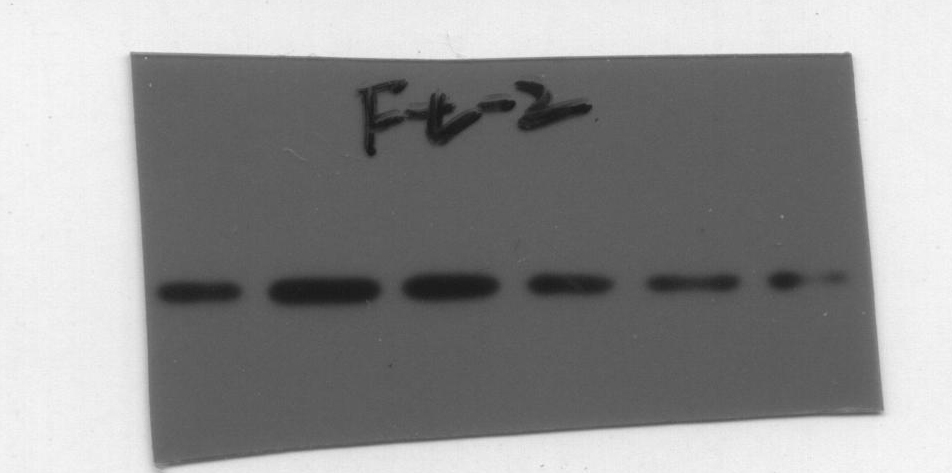

Supplement: Supplemental Information 1 [file peerj-10-13592-s001.zip › Raw data/Fig.10/Ft (2).jpg]

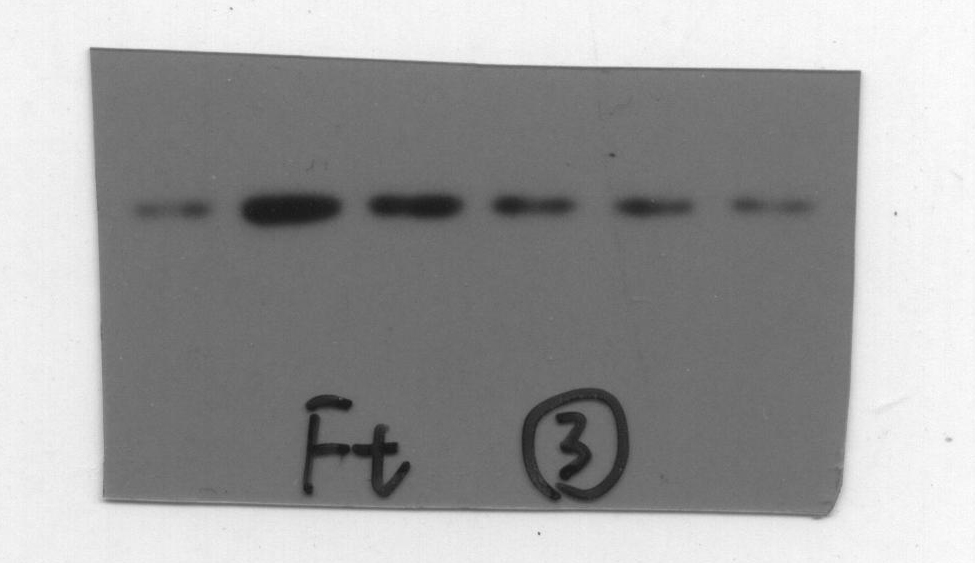

Supplement: Supplemental Information 1 [file peerj-10-13592-s001.zip › Raw data/Fig.10/Ft (3).jpg]

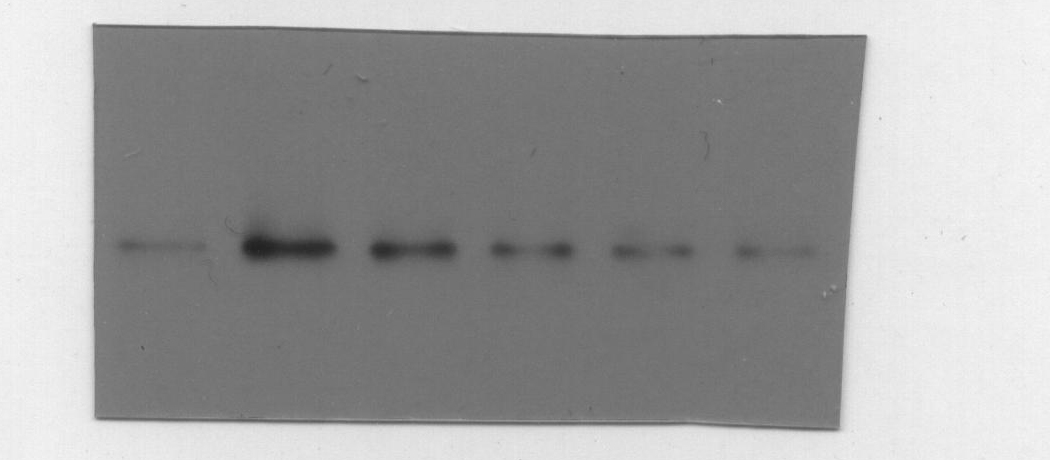

Supplement: Supplemental Information 1 [file peerj-10-13592-s001.zip › Raw data/Fig.10/Hepcidin 1.jpg]

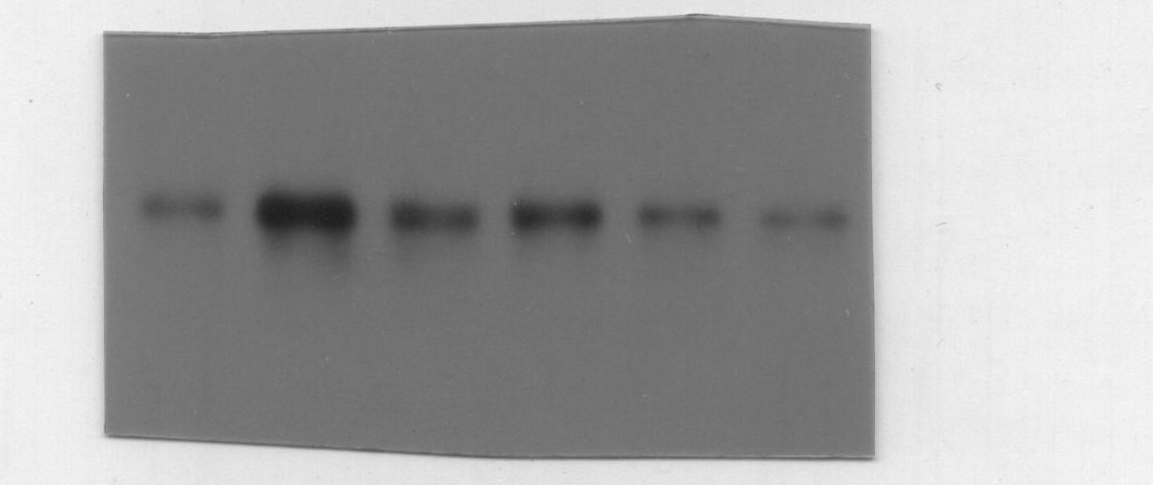

Supplement: Supplemental Information 1 [file peerj-10-13592-s001.zip › Raw data/Fig.10/Hepcidin 2.jpg]

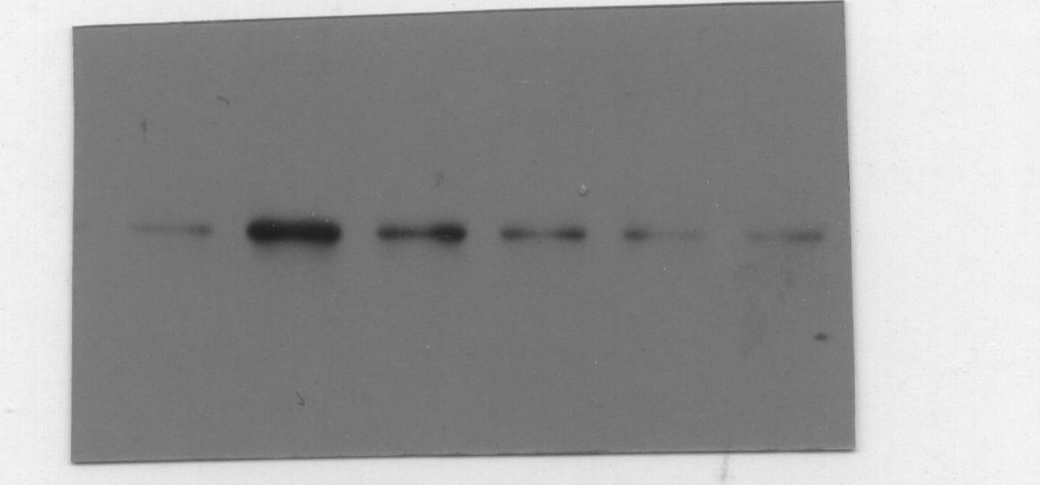

Supplement: Supplemental Information 1 [file peerj-10-13592-s001.zip › Raw data/Fig.10/Hepcidin 3.jpg]

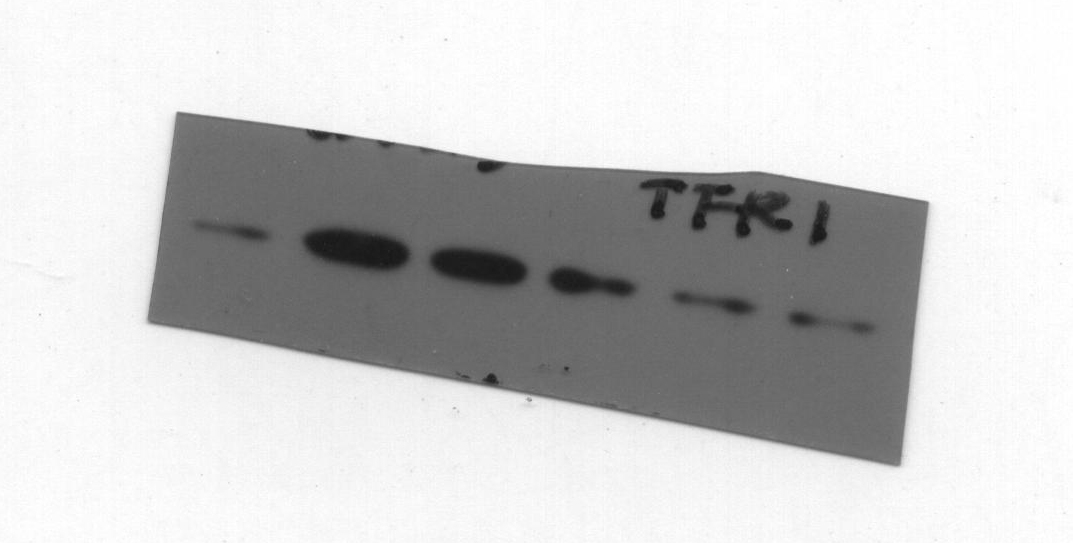

Supplement: Supplemental Information 1 [file peerj-10-13592-s001.zip › Raw data/Fig.10/TFR2 ú¿1ú⌐.jpg]

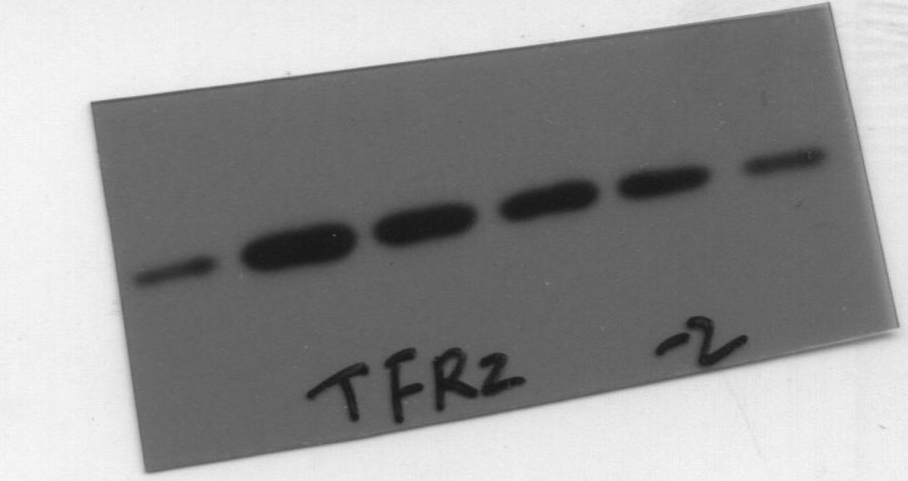

Supplement: Supplemental Information 1 [file peerj-10-13592-s001.zip › Raw data/Fig.10/TFR2 ú¿2ú⌐.jpg]

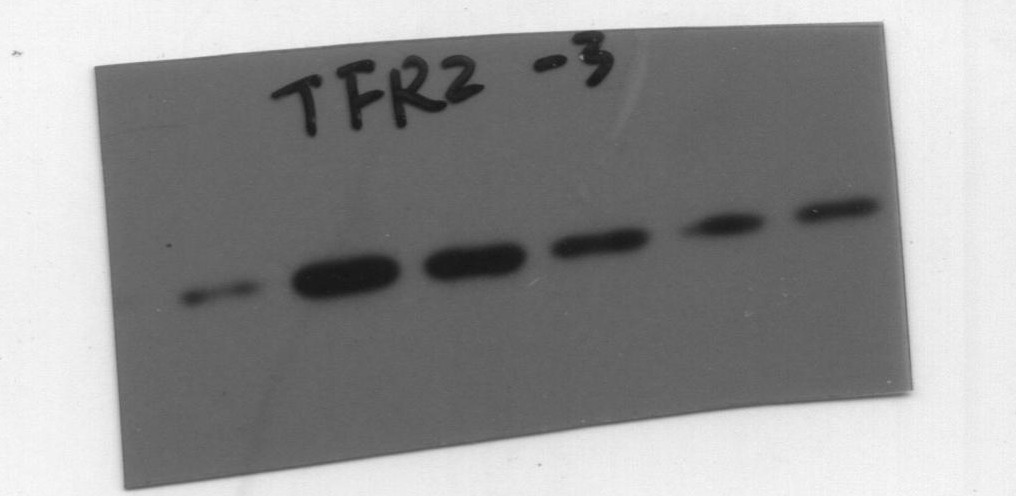

Supplement: Supplemental Information 1 [file peerj-10-13592-s001.zip › Raw data/Fig.10/TFR2 ú¿3ú⌐.jpg]

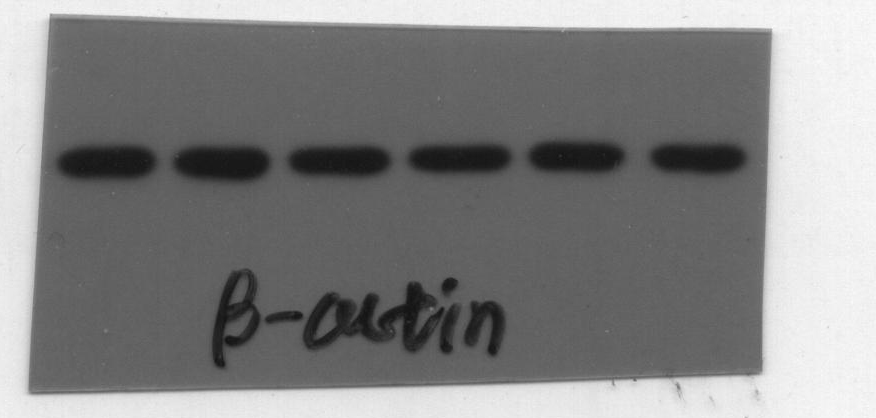

Supplement: Supplemental Information 1 [file peerj-10-13592-s001.zip › Raw data/Fig.10/a┬-actin ú¿1ú⌐.jpg]

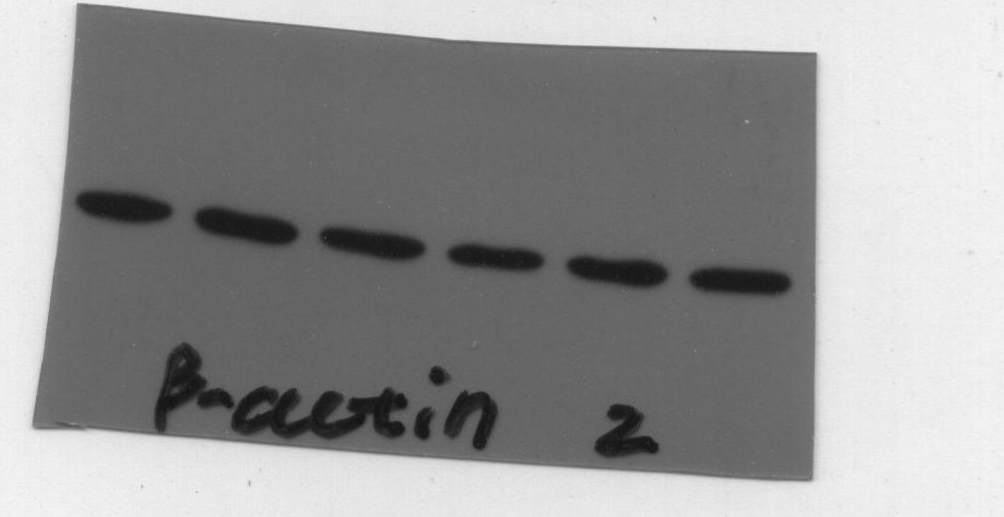

Supplement: Supplemental Information 1 [file peerj-10-13592-s001.zip › Raw data/Fig.10/a┬-actin ú¿2ú⌐.jpg]

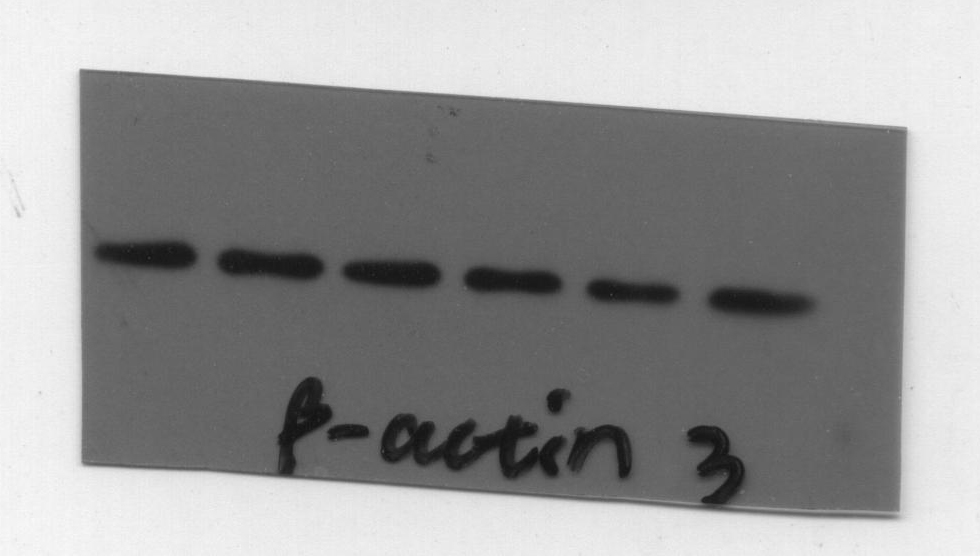

Supplement: Supplemental Information 1 [file peerj-10-13592-s001.zip › Raw data/Fig.10/a┬-actin ú¿3ú⌐.jpg]

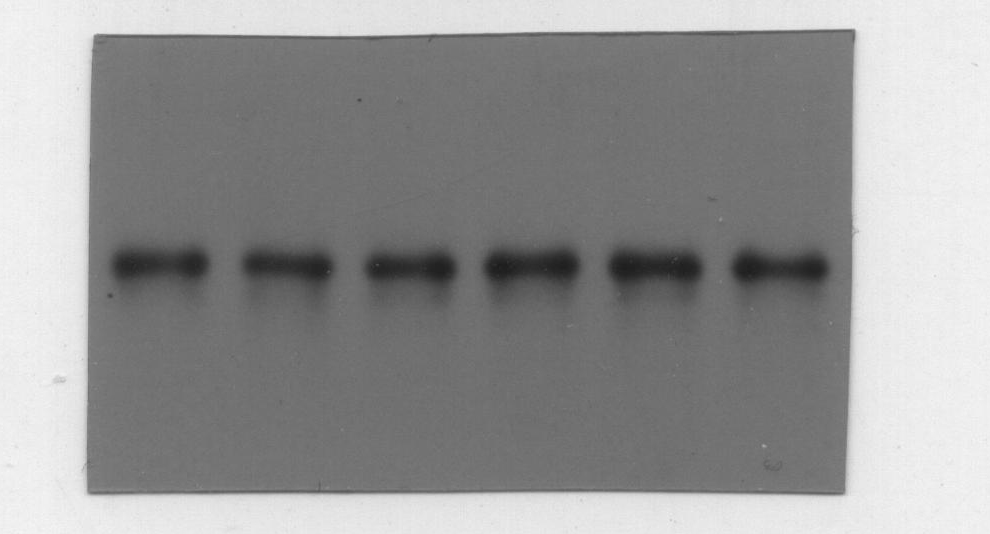

Supplement: Supplemental Information 1 [file peerj-10-13592-s001.zip › Raw data/Fig.10/a┬-actin 1.jpg]

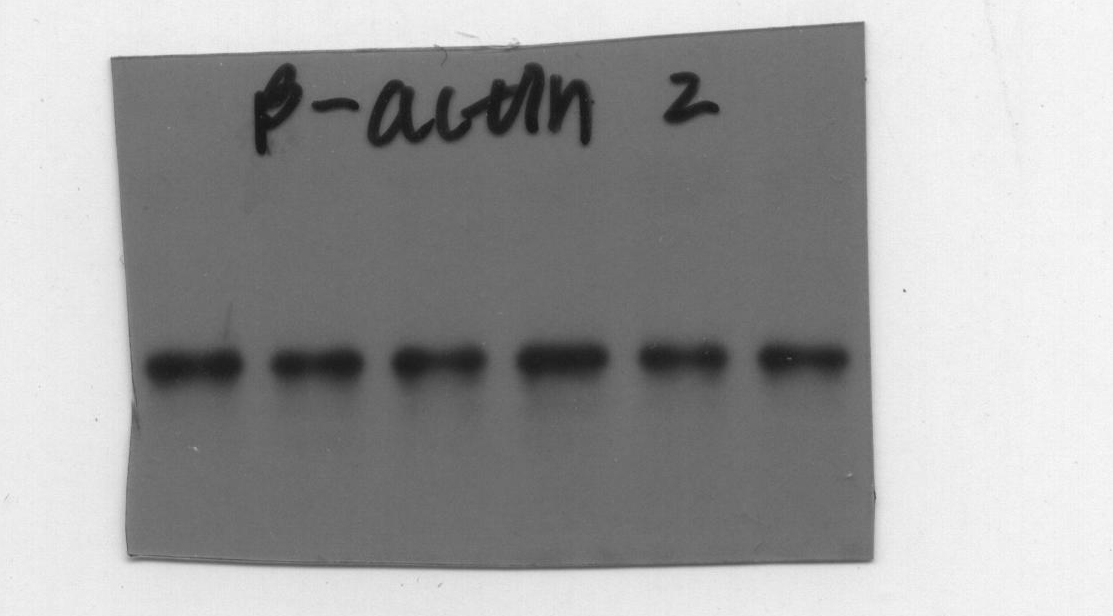

Supplement: Supplemental Information 1 [file peerj-10-13592-s001.zip › Raw data/Fig.10/a┬-actin 2.jpg]

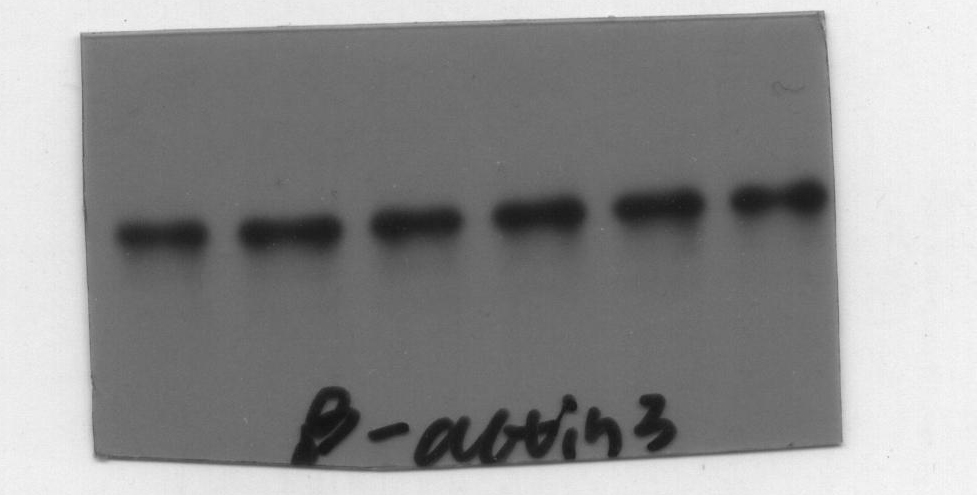

Supplement: Supplemental Information 1 [file peerj-10-13592-s001.zip › Raw data/Fig.10/a┬-actin 3.jpg]

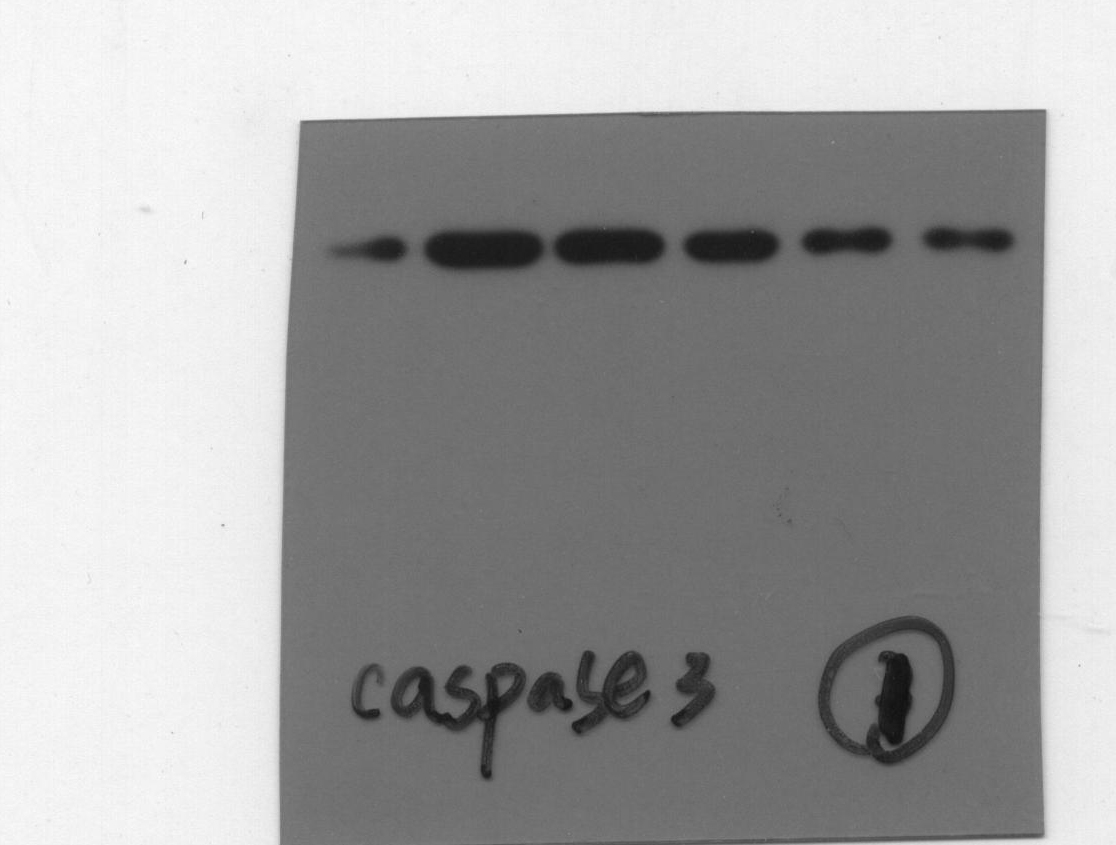

Supplement: Supplemental Information 1 [file peerj-10-13592-s001.zip › Raw data/Fig.7/Caspase3 ú¿1ú⌐.jpg]

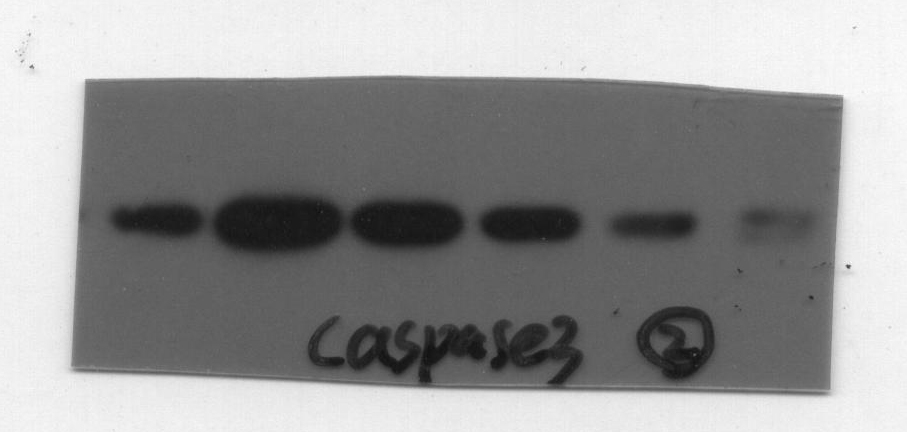

Supplement: Supplemental Information 1 [file peerj-10-13592-s001.zip › Raw data/Fig.7/Caspase3 ú¿2ú⌐.jpg]

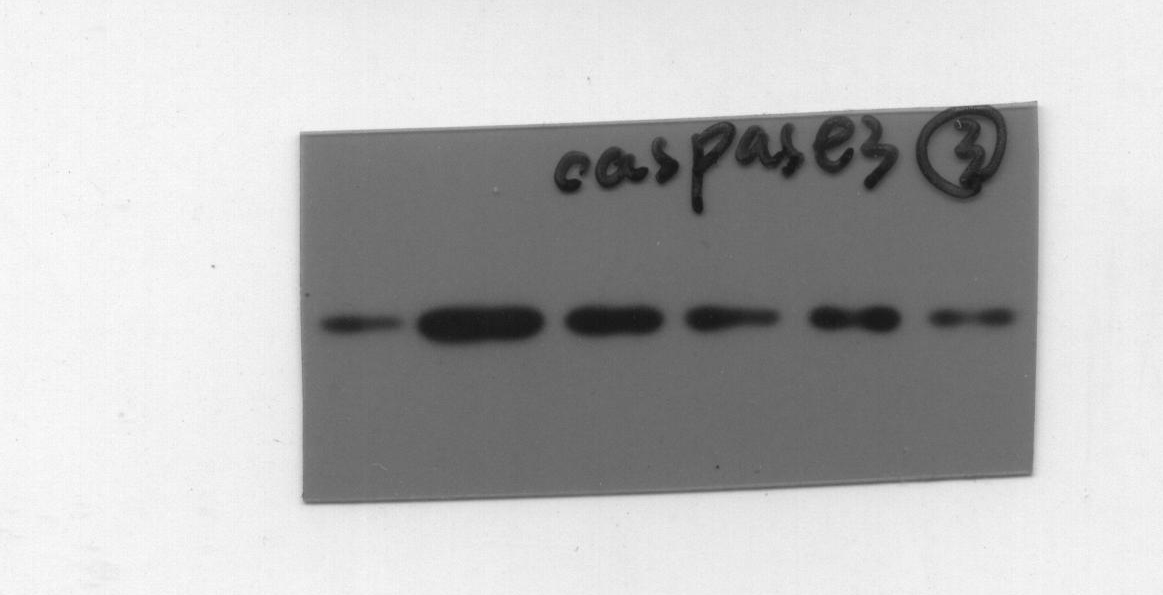

Supplement: Supplemental Information 1 [file peerj-10-13592-s001.zip › Raw data/Fig.7/Caspase3 ú¿3ú⌐.jpg]

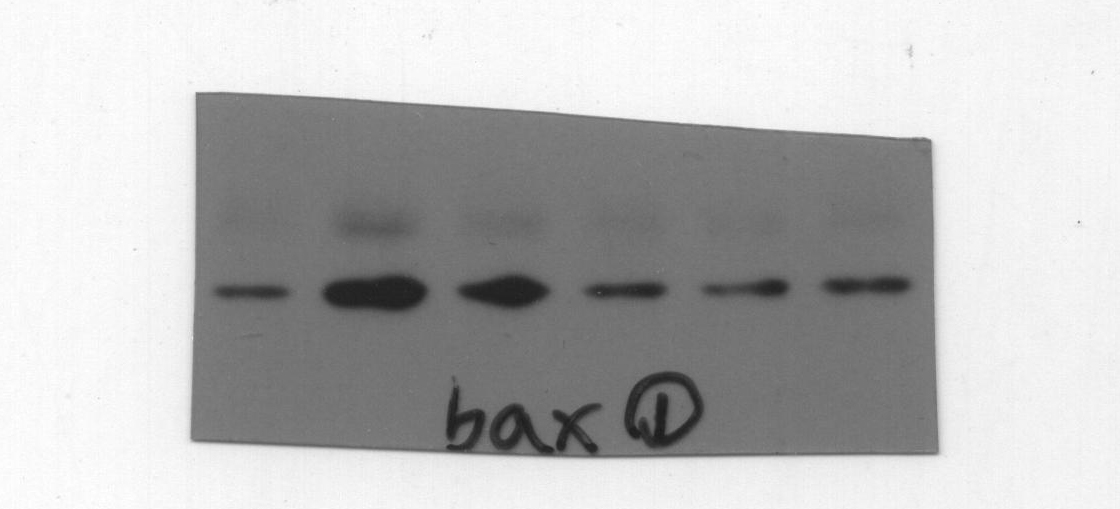

Supplement: Supplemental Information 1 [file peerj-10-13592-s001.zip › Raw data/Fig.7/bax ú¿1ú⌐.jpg]

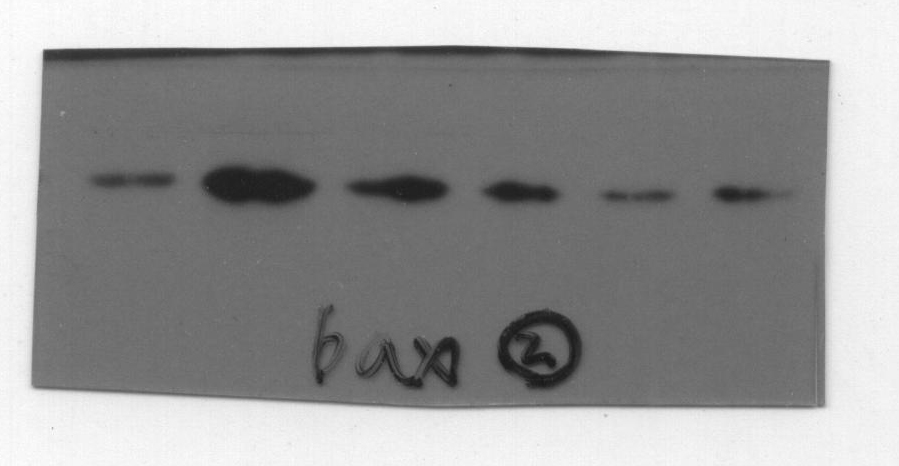

Supplement: Supplemental Information 1 [file peerj-10-13592-s001.zip › Raw data/Fig.7/bax ú¿2ú⌐.jpg]

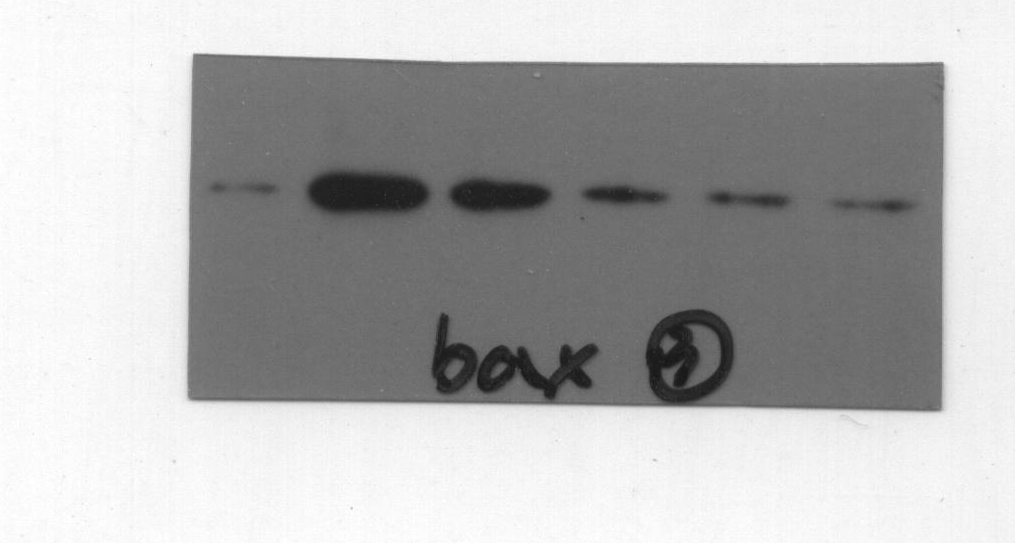

Supplement: Supplemental Information 1 [file peerj-10-13592-s001.zip › Raw data/Fig.7/bax ú¿3ú⌐.jpg]

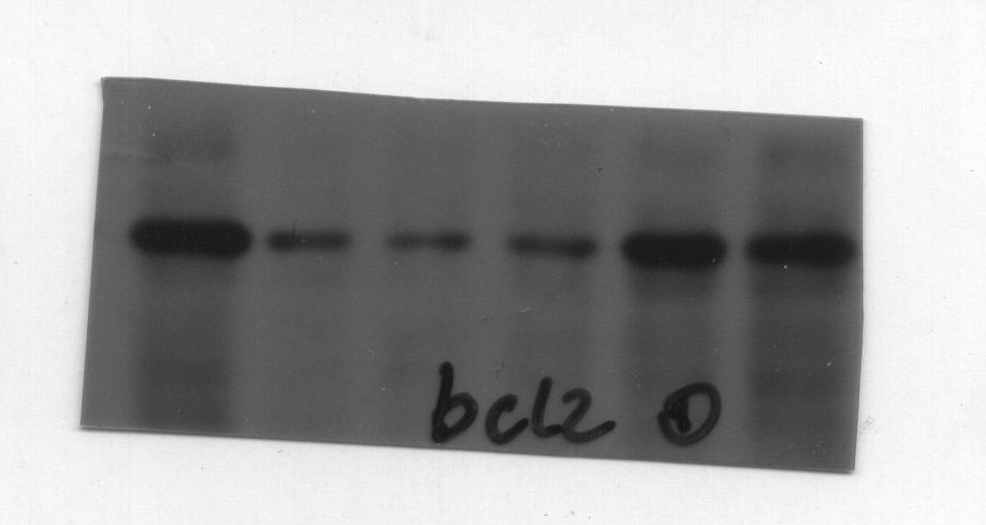

Supplement: Supplemental Information 1 [file peerj-10-13592-s001.zip › Raw data/Fig.7/bcl2 ú¿1ú⌐.jpg]

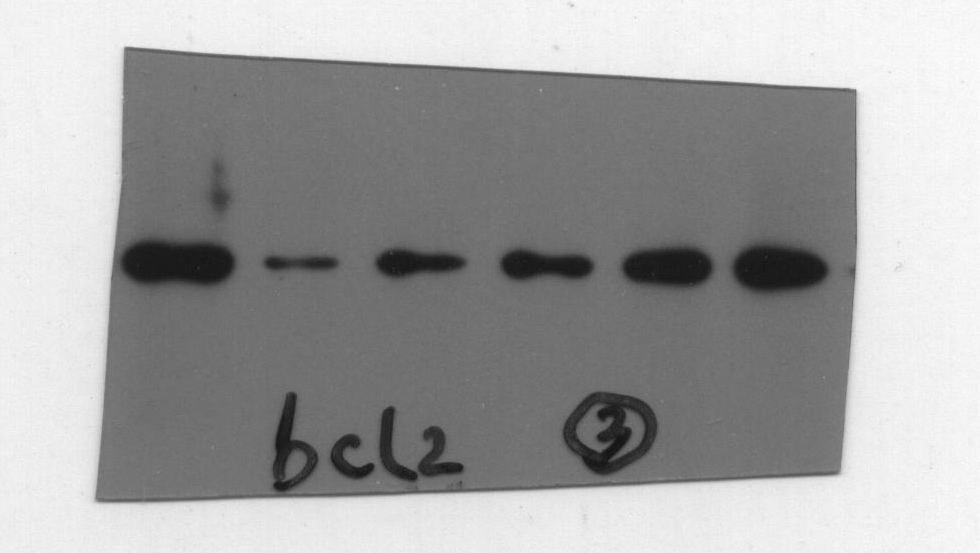

Supplement: Supplemental Information 1 [file peerj-10-13592-s001.zip › Raw data/Fig.7/bcl2 ú¿3ú⌐.jpg]

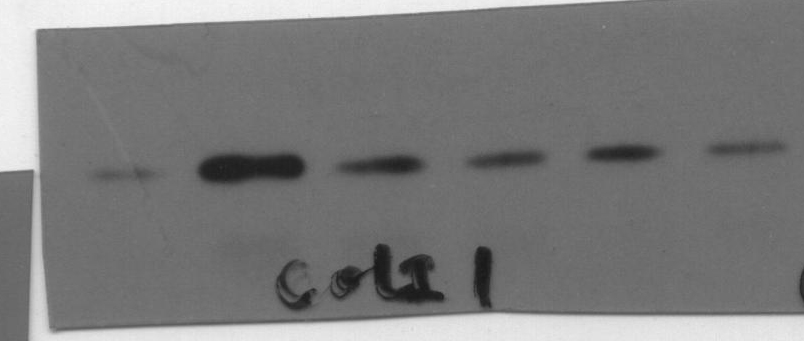

Supplement: Supplemental Information 1 [file peerj-10-13592-s001.zip › Raw data/Fig.9/Col-I (1).jpg]

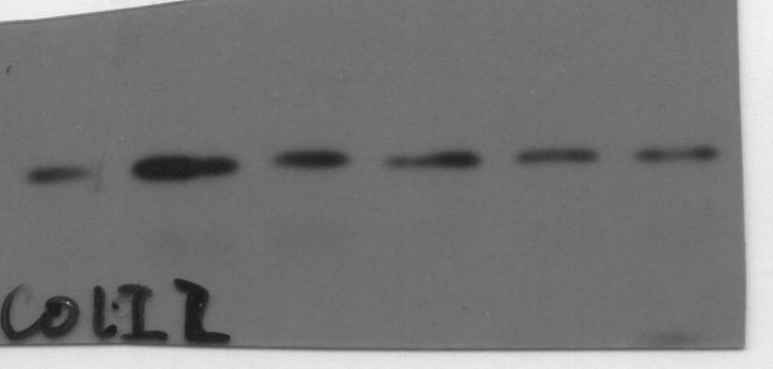

Supplement: Supplemental Information 1 [file peerj-10-13592-s001.zip › Raw data/Fig.9/Col-I (2).jpg]

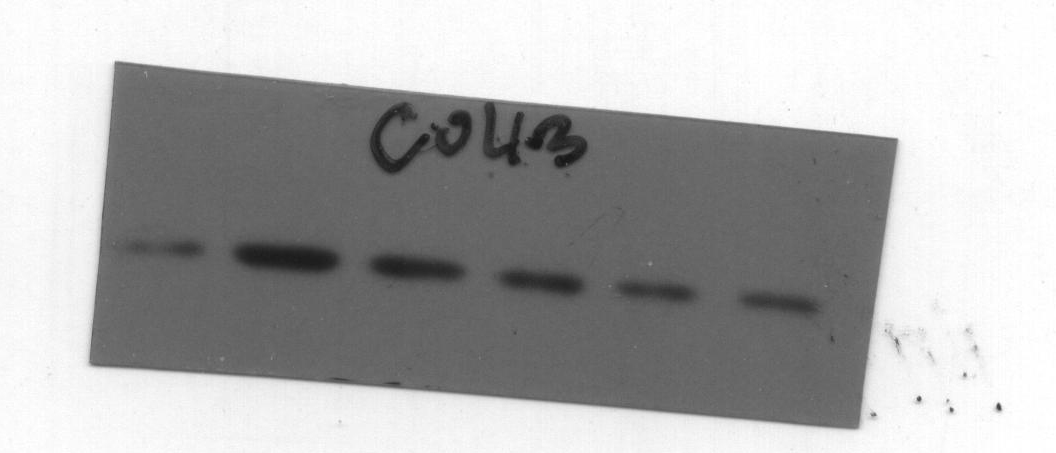

Supplement: Supplemental Information 1 [file peerj-10-13592-s001.zip › Raw data/Fig.9/Col-I (3).jpg]

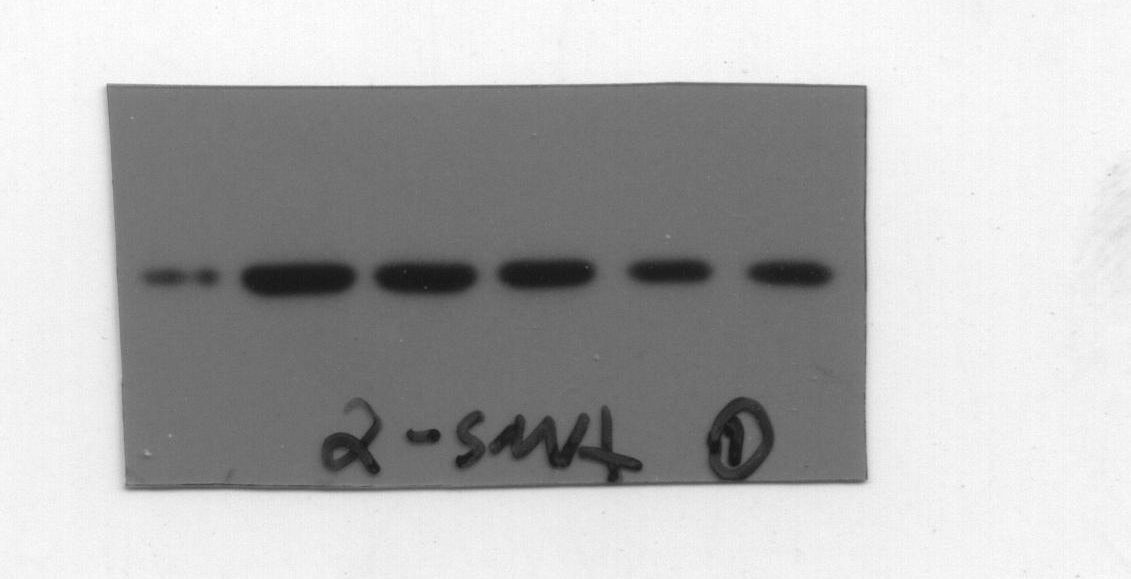

Supplement: Supplemental Information 1 [file peerj-10-13592-s001.zip › Raw data/Fig.9/a┴-SMA ú¿1ú⌐.jpg]

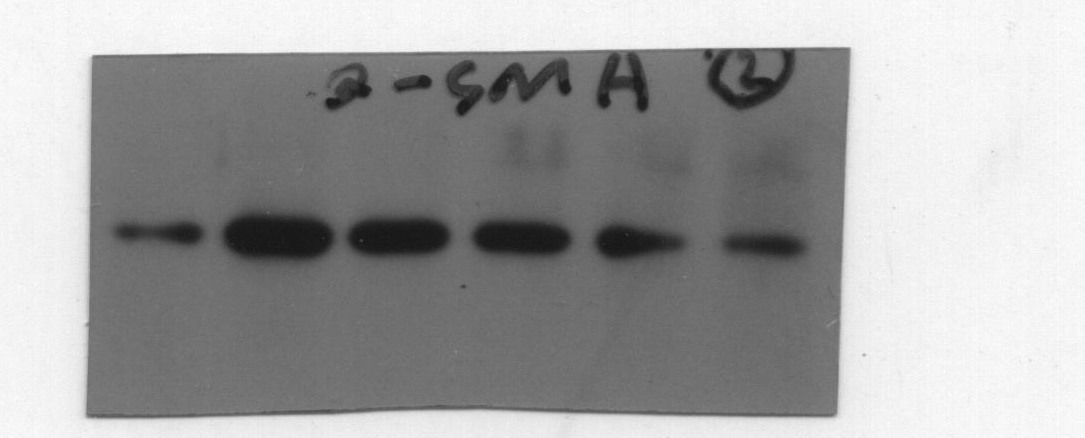

Supplement: Supplemental Information 1 [file peerj-10-13592-s001.zip › Raw data/Fig.9/a┴-SMA ú¿2ú⌐.jpg]

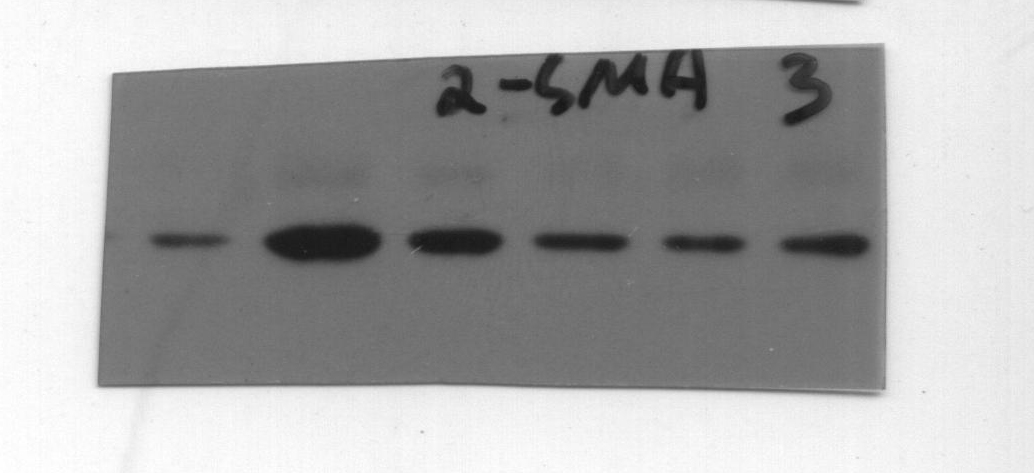

Supplement: Supplemental Information 1 [file peerj-10-13592-s001.zip › Raw data/Fig.9/a┴-SMA ú¿3ú⌐.jpg]
